# Frog Skin Peptides: Nature’s Dual-Action Weapons Against Infection and Cancer

**DOI:** 10.3390/antibiotics15030324

**Published:** 2026-03-23

**Authors:** Eleonora Grisard, Carlo Vetrano, Ali Benour, Eeva Tortellini, Dania Al Ismail, Giacomo Cappella, Bruno Casciaro, Maria Luisa Mangoni, Milena Mechkarska

**Affiliations:** 1 Department of Biochemical Sciences, Laboratory Affiliated to Istituto Pasteur Italia-Fondazione Cenci Bolognetti, Sapienza University of Rome, p.le Aldo Moro 5, 00185 Rome, Italy; eleonora.grisard@uniroma1.it (E.G.); carlo.vetrano@uniroma1.it (C.V.); eeva.tortellini@uniroma1.it (E.T.); dania.alismail@uniroma1.it (D.A.I.); giacomo.cappella@uniroma1.it (G.C.); 2 Department of Surgery, RCSI University of Medicine and Health Sciences, D02 YN77 Dublin, Ireland; alibenour20@rcsi.ie; 3 Department of Life Sciences, Faculty of Science and Technology, The University of the West Indies, St. Augustine Campus, St. Augustine, Trinidad and Tobago

**Keywords:** frog skin peptides, antimicrobial peptides, anticancer peptides, dual-function peptides, bioactive peptides, membrane disruption, apoptosis, peptide therapeutics, anuran skin secretions

## Abstract

The rise of antimicrobial resistance and the global burden of cancer demand innovative therapeutic strategies. Frog skin secretions offer a rich source of bioactive peptides, some of which exhibit remarkable dual functionality—potent antimicrobial activity coupled with selective anticancer effects. This review highlights frog skin-derived peptides that bridge the gap between antimicrobial and anticancer therapeutics, emphasizing their structural diversity, mechanisms of action, and translational potential. A comprehensive literature search was conducted to identify peptides isolated from diverse anuran species, with emphasis on studies reporting structural features, activity against Gram-positive and Gram-negative bacteria, including multidrug resistant clinical isolates, anticancer effects, and underlying molecular mechanisms of cytotoxicity. Peptides such as dermaseptins, temporins, and brevinins disrupt microbial membranes while triggering apoptosis or necrosis in cancer cells. Key physicochemical characteristics, including net positive charge, amphipathicity, and α-helical conformation, contribute to their dual functionality. Recent advances in peptide engineering and delivery have improved stability, selectivity, and therapeutic efficacy, enhancing the clinical prospects of these naturally occurring bioactive molecules. Frog skin peptides represent promising candidates for the development of next-generation antimicrobial and anticancer therapeutics.

## 1. Introduction

The COVID-19 pandemic has made the world acutely aware of the impact of global health emergencies [1]. Yet, another ongoing crisis—often described as a “silent pandemic”—is projected to cause 10 million deaths per year by 2050: antimicrobial resistance (AMR) [2]. The extensive use of antibiotics in clinical settings and in agriculture has accelerated the emergence and environmental spread of multidrug-resistant (MDR) bacteria. In parallel, the discovery of new antibiotics has markedly declined, creating a critical threat to global health [3,4,5]. At the same time, cancer-related mortality is also expected to rise dramatically. By 2050, cancer is projected to cause 18.5 million deaths, representing an 89.7% increase compared with the 2022 estimate of 9.7 million deaths [6]. Cancer is not a single disease but rather a heterogeneous group of diseases comprising more than 250 clinico-pathological types and thousands of described neoplastic variants [7,8]. In this increasingly complex landscape, the links between microbial infections—caused by viruses, bacteria, and fungi—and tumorigenesis have become more evident. Major infectious contributors to cancer development include *Helicobacter pylori* (5%), human papillomaviruses (HPV) (5%), hepatitis B and C viruses (HBV, HCV) (5%), Epstein–Barr virus (EBV) (1%), and HIV together with human herpesvirus (HSV) (1%). Moreover, chemotherapy can profoundly disrupt the host microbiome, increasing susceptibility to infections and potentially promoting cancer development [9]. Persistent microbial infections that induce chronic inflammation are now recognized as key drivers of carcinogenesis in affected tissues [10]. Given the dangerous interplay between microbial infection and cancer development, discovering compounds with dual antibacterial and anticancer properties is a highly valuable strategy—potentially reducing toxicity, limiting the need for multiple drugs, and mitigating resistance.

Antimicrobial peptides (AMPs) represent a promising class of such therapeutics. AMPs are gene-encoded bioactive peptides produced by virtually all living organisms. Synthesized as pre-propeptides, they are processed into their active form through proteolytic cleavage [11]. They are typically cationic, short (10–50 amino acids), and amphipathic, and they exert antimicrobial activity primarily by interacting with and disrupting microbial membranes [12]. Nonetheless, AMPs also act through a variety of alternative mechanisms: for instance, proline-rich AMPs inhibit protein synthesis by binding to ribosomes [13]; others interfere with membrane protein functions, such as the LPS transport system [14]; and compounds like darobactin target components of the β-barrel assembly machinery [15]. This mechanistic diversity reduces the likelihood of resistance development [16]. Beyond their antimicrobial role, AMPs often possess a wide array of biological functions; therefore, they are referred to as “host defense peptides” (HDPs) [17]. They can also act as drug-delivery vectors, signaling molecules, contraceptive agents, immunomodulators, mitogenic factors, and, importantly, antitumor agents [18].

## 2. Amphibian Skin Peptides: Blueprint for Development of Antimicrobial and Anticancer Therapeutics

Frog skin secretions have captivated researchers for decades as a natural pharmacy of bioactive molecules. Among their most fascinating components are the AMPs, small, cationic, and amphipathic molecules that serve as the first line of defense in amphibian immunity. The story began in the 1980s, when Michael Zasloff’s discovery of magainins from the African clawed frog *Xenopus laevis* unveiled a new class of natural antibiotics capable of killing a wide range of pathogens [19]. Since then, hundreds of structurally diverse AMPs have been isolated from frog species across the families Ranidae, Hylidae, and Phyllomedusidae [20,21]. Their remarkable structural plasticity—ranging from α-helices to β-sheets and looped forms—has made them invaluable templates for designing novel antimicrobial agents at a time when infections caused by MDR bacteria pose a mounting global threat [22].

In recent years, a new role has emerged for these peptides: several frog skin AMPs also exhibit potent anticancer activity. Peptides such as dermaseptins, temporins, and brevinins can selectively destroy cancer cells while sparing healthy ones, exploiting differences in membrane charge and composition [23,24]. For instance, dermaseptin-B2 (from *Phyllomedusa bicolor*) not only shows broad antimicrobial effects but also inhibits the growth of human prostate and breast cancer cells by inducing apoptosis and disrupting cellular membranes [24,25]. Likewise, temporin-1CEa (from *Rana chensinensis*) and brevinin-2R (from *Pelophylax ridibundus*) have demonstrated anticancer activity through mitochondrial damage and caspase activation [26]. The physicochemical features that empower these peptides—cationicity, amphipathicity, and α-helical conformation—enable them to interact with negatively charged surfaces common to both microbial and tumor cells. This dual functionality places frog-derived AMPs at the crossroads of antimicrobial and anticancer drug discovery, positioning them as natural blueprints for multifunctional therapeutics in an era of widespread hospital and community-associated infections, antimicrobial resistance and oncologic challenges.

A novel and rapidly expanding approach has emerged that leverages artificial intelligence (AI) and machine learning (ML) models to identify recurring sequence–structure–function relationships, thereby accelerating the discovery of peptides as both antimicrobial and anticancer therapeutic leads. Key physicochemical features, such as those discussed earlier, which underpin selective membrane disruption in microbial and cancer cell membranes, can be effectively predicted using ML models trained on large datasets of AMPs and anticancer peptides [27,28]. These AI-driven strategies enable rapid in silico screening and rational peptide design, allowing prioritisation of candidates with high predicted efficacy and reduced host toxicity prior to costly synthesis and biological evaluation. Most published studies report AI-guided peptide discovery workflows that employ computational tools typically focused on individual prediction tasks—either antimicrobial or anticancer activity. While such studies establish a strong foundation for dual-function peptide discovery, combined experimental validation of peptides predicted to possess both activities remains an emerging area. To date, most validation studies are limited to in vitro evaluation of antimicrobial activity against multiple bacterial species and anticancer effects in tumor-derived cell lines [29], whereas direct in vivo validation of AI-designed peptides with dual antimicrobial and anticancer activity in animal models remains rare. Consequently, ML/AI-based prediction and design strategies were not a central focus of this article.

This review summarizes the experimentally derived data available to date on selected AMPs with dual activity, including their names and primary structures, the frog species from which they were isolated [30], and their anticancer and antibacterial activities as per the original publications (Table 1). Anticancer activity is reported as lethal concentration 50% (LC_50_) or inhibitory concentration 50% (IC_50_) values for cancer cells, while antibacterial activity is expressed as minimal inhibitory concentrations (MICs) or lethal concentrations (LCs) for bacterial growth. We then focus in detail on the principal physicochemical and molecular mechanisms underlying their anticancer activity (Section 2.1), followed by an analysis of the functional consequences of AMP treatment on tumor cell behavior (Section 2.2), conserved mode of AMP action across species (Section 2.3), and translational implications and safety concerns (Section 2.4).

### 2.1. Mechanisms of Anticancer Action

**Figure 1:**
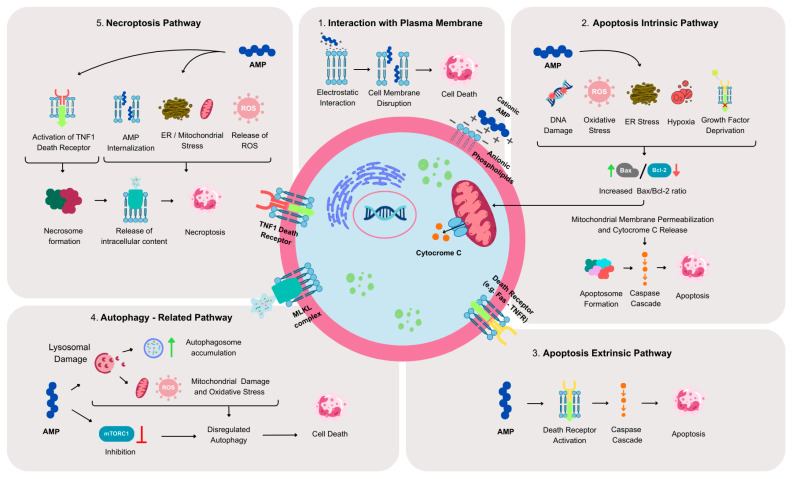
Schematic overview of the principal cellular and molecular mechanisms for elimination of a tumor cell (center) by AMPs: (**1**) direct cytolysis via disruption of the plasma membrane (pink circle); (**2**) activation of the intrinsic (mitochondria-mediated) apoptotic pathway; (**3**) activation of the extrinsic (death receptor-mediated) apoptotic pathway; (**4**) induction of cell death through autophagy; and (**5**) initiation of necroptosis.

Several frog skin-derived AMPs have been evaluated for both antimicrobial and anticancer activities. Their anticancer potential has been investigated predominantly in vitro using a wide range of human tumor-derived cell lines and, to a lesser extent, in vivo in murine cancer models. The principal molecular mechanisms underlying the anticancer activity of AMPs are summarized (Figure 1) and are discussed in detail in the following sections.

#### 2.1.1. Cytolysis Mediated by Physical Interaction with the Tumor Cell Plasma Membrane

Most AMPs are enriched in positively charged amino acids—arginine, lysine, and histidine—which confer a strong cationic character. This property underlies their antimicrobial activity by enabling electrostatic interactions with negatively charged bacterial membranes [155]. In contrast, plasma membranes of normal eukaryotic cells are dominated by neutral phospholipids such as phosphatidylcholine. Many tumor cells, however, undergo compositional remodeling that results in an increased abundance of negatively charged lipids—including phosphatidylserine, phosphatidic acid, and altered cholesterol distribution—resulting in a predominantly more anionic membrane surface [156]. As a consequence, cationic AMPs from both vertebrate and invertebrate organisms often display selective cytolytic activity toward cancer cells via mechanisms analogous to those used against bacterial membranes [157].

Two examples of such peptides, bombinin H4 and temporin A, were recently tested on non-small cell lung cancer (NSCLC) cell lines and normal bronchial epithelial cells [43]. Both peptides exhibited selective cytotoxicity toward NSCLC cells, though to different degrees. Lipidomic profiling revealed significant differences in membrane phospholipid composition between normal and tumor cells, with NSCLC cells displaying higher levels of negatively charged phospholipids. These findings support the idea that the selective anticancer action of cationic AMPs is linked, at least in part, to preferential binding to and disruption of anionic tumor membranes. Bombinin H4 also inhibited the growth of *S. epidermidis* strains 1457 and 5179-R1, highlighting its dual antimicrobial and anticancer functionality [43].

However, membrane-mediated tumor killing is not restricted only to cationic AMPs. The anionic amphibian peptide maximin-H5, active against Gram-positive *S. aureus* [122], selectively killed human glioblastoma cells while sparing normal glial cells. The potent cytotoxicity is attributed to its ability to adopt an α-helical conformation in proximity to the anionic glioblastoma membrane, leading to non-membranolytic, yet selective, tumor cell death [120].

Many anticancer AMPs from both vertebrate and invertebrate organisms exhibit membranolytic activity [157]. The most common assay used to evaluate plasma membrane integrity is the lactate dehydrogenase (LDH) release test, which detects extracellular LDH as an indicator of membrane damage [158]. For frog-skinderived anticancer AMPs, membrane lysis is a frequently reported consequence of peptide treatment.

In studies where AMPs induce tumor cell death through defined intracellular cell-death pathways (see sections below), LDH release is often only one among several functional assays [88,134,150]. Due to the inherent limitations of in vitro experiments, which cannot easily resolve the chronological order of events following peptide exposure, it remains unclear whether membrane disruption is a downstream consequence of programmed cell death or the initiating event that triggers such pathways.

In other studies, LDH release represents the sole measure of membrane integrity, accompanied only by general cytotoxicity assays [87,90,91,129,135,143,159]. This makes it even more challenging to determine whether membrane permeabilization arises as part of a defined cellular/molecular death program or as a primary effect of a direct AMP–membrane interaction.

Notably, all of these peptides also exhibit broad-spectrum antimicrobial activity, strengthening the hypothesis that membrane disruption may be a conserved mechanism across microbial and cancer targets. The antimicrobial spectra are reported mostly for strains of the ESKAPE+ pathogens (highly virulent MDR bacteria responsible for the majority of hospital-acquired infections worldwide, including: *Enterococcus faecium*, *Staphylococcus aureus*, *Klebsiella pneumoniae*, *Acinetobacter baumannii*, *Pseudomonas aeruginosa*, and *Enterobacter* spp.), as well as for MRSA and various bacterial MDR clinical isolates [87,90,91,110,129,135,140,159].

#### 2.1.2. Activation of Cell-Death Pathways

Most amphibian AMPs with anticancer properties described in this review exert their effects through the direct or indirect activation of specific cell-death programs. The predominant pathways engaged by these peptides include: (i) the intrinsic apoptotic pathway, (ii) the extrinsic (death receptor-mediated) apoptotic pathway, (iii) autophagy-related cell-death mechanisms, and (iv) the necroptosis pathway (see also Figure 1).

(i) Apoptosis Intrinsic Pathway (Mitochondrial Pathway)

The intrinsic, or mitochondrial, apoptotic pathway is activated by internal cellular stress signals, including DNA damage, hypoxia, ER stress, oxidative stress, or deprivation of growth factors. These stimuli are sensed primarily by Bcl-2 family proteins, which regulate mitochondrial membrane integrity [160]. Activation of the pro-apoptotic member Bax promotes mitochondrial outer membrane permeabilization, cytochrome-c release, and initiation of the apoptotic cascade; thus, an increased Bax/Bcl-2 ratio is widely used as an indicator of mitochondrial apoptosis activation [161].

Temporin-SHf, a peptide with strong potency against *E. coli*, was shown to selectively induce mitochondrial apoptosis in lung cancer cells [150]. Initial 3-(4,5-dimethylthiazol-2-yl)-2,5-diphenyltetrazolium bromide (MTT) assays revealed selective toxicity toward various tumor cell lines (lung, breast, prostate, liver) but not toward normal endothelial cells. A549 lung cancer cells, identified as the most sensitive, displayed a dose-dependent increase in p53 and Bax expression following treatment, leading to elevation of the Bax/Bcl-2 ratio. The mitochondrial apoptotic program was further supported by increased levels of initiator and effector molecules—including total and cleaved caspase-9, -8, -3, and PARP—as well as chromatin condensation and fragmentation. Temporin-SHf also caused membranolytic effects (measured by LDH release), reduced tumor cell growth in soft agar, and impaired tumor-cell migration in a pseudo-wound-healing assay, highlighting its broad functional impact on cancer cell behavior [150].

Hymenochirin-1B, active against several Gram-positive and Gram-negative bacteria [105,106,162], similarly exhibited selective toxicity toward hepatoma and lung carcinoma cells by MTT assay, with lung cancer cells again being the most sensitive [105]. Unlike temporin-SHf, hymenochirin-1B did not induce membrane lysis by LDH assay. Instead, it triggered G0/G1 cell-cycle arrest and apoptosis (Annexin V/PI staining) through direct interaction with mitochondria. This was accompanied by mitochondrial membrane potential dissipation, elevated ROS production, an increased Bax/Bcl-2 ratio, and enhanced expression of cleaved caspase-3. Functionally, hymenochirin-1B reduced tumor-cell migration and clonogenicity.

Dermaseptin-PS1, moderately active against *S. aureus*, *E. coli*, and *C. albicans*, was evaluated in several tumor cell lines and in normal endothelial cells [88]. At low concentrations (10^−6^ M), it showed selective toxicity toward U215MG glioblastoma cells while sparing normal cells. At this concentration, LDH release was minimal, yet caspase-3 cleavage was evident, indicating early activation of apoptosis. This cleavage was fully prevented by a pan-caspase inhibitor, confirming the involvement of caspases. Expression analyses revealed upregulation of multiple pro-apoptotic markers—including Bax—and increased Bax/Bcl-2 ratio, p53 phosphorylation, and cytosolic cytochrome-c release. Autophagy-related and extrinsic apoptotic pathways were excluded, demonstrating that dermaseptin-PS1 acts specifically through mitochondria-dependent apoptosis [88].

Ranatuerin-2PLx, particularly active toward *S. aureus* and *E. coli*, was tested across a range of tumor and normal cell lines [134]. At high concentrations, it was selectively toxic to tumor cells—especially PC3 prostate cancer cells—while sparing normal cells. In PC3 cells, the peptide caused membrane lysis (measured by LDH release) and induced apoptosis as demonstrated by Annexin V staining. Increased caspase-3 activity suggested involvement of an intrinsic apoptotic pathway, although it is shared by both intrinsic and extrinsic pathways; therefore, additional analyses would be needed to define the mechanism conclusively.

In some cases, frog-derived AMPs exhibit cytotoxic profiles compatible with mitochondrial apoptosis, even when no specific molecular analyses were performed. For example, temporin-La and palustrin-Ca were tested on cervical, gastric, and hepatic cancer cells [126]. Both peptides exhibited strong cytotoxicity in MTT assays. Transmission electron microscopy (TEM) analysis in HeLa cells revealed membrane damage, cytoplasmic leakage, and blurred cell boundaries, along with conspicuous mitochondrial vacuolization and loss of cristae—features consistent with mitochondrial dysfunction and suggestive of intrinsic apoptosis. Both peptides were more active against Gram-positive than Gram-negative bacteria.

The AMP pentadactylin, active against Gram-negative and Gram-positive bacteria [127], showed selective toxicity toward B16-F10 melanoma cells [128]. Cytological analyses demonstrated membrane disruption, formation of cellular protrusions, and altered nuclear morphology. The peptide also induced S-phase cell-cycle arrest, mitochondrial membrane potential loss, and DNA fragmentation—strong indicators of mitochondrial apoptosis.

(ii) Apoptosis Extrinsic Pathway (Death Receptor Pathway)

The extrinsic pathway of apoptosis is initiated by extracellular signals, typically through the binding of death ligands—such as FasL, TNF-α, or TRAIL—to their cognate receptors on the plasma membrane. This receptor-mediated mechanism plays a central role in immune regulation and in the removal of damaged, infected, or superfluous cells [160].

The peptides caerin 1.1 and caerin 1.9, exhibit broad spectrum activity against Gram-negative and Gram-positive bacteria, including MRSA strains and in vivo antimicrobial effect using murine MRSA skin infection model [68,163]. When tested on HeLa cervical cancer cells, both peptides inhibited cell proliferation as determined by MTT assay [63]. Microscopy-based studies showed that the peptides interact directly with the plasma membrane and become internalized, localizing to perinuclear regions within the first 5 min of treatment.

Comprehensive proteomic profiling of HeLa cells treated with caerin 1.1, caerin 1.9, or a combination of the two revealed substantial modulation of cellular pathways. Pathway-enrichment analyses indicated that caerin 1.1—alone or combined with caerin 1.9—upregulated biological processes related to mRNA stability and the cellular response to unfolded or misfolded proteins. Most importantly, both peptides significantly enhanced pathways linked to programmed cell death, leading to upregulation of proteins associated with apoptotic signaling. In particular, caerin 1.1 or 1.9 treatment selectively increased the apoptosis-related branch of the TNF-α signaling pathway, which was experimentally validated by elevated expression of caspase-3 and -9, consistent with activation of the extrinsic apoptosis pathway [63].

AMP-induced modulation of EGFR signaling has been described in non-tumor contexts, especially during wound healing [164,165]. Interestingly, in HeLa cancer cells, caerin peptides had the opposite effect: they downregulated the EGFR1 pathway, as confirmed by a dose-dependent reduction of PI3K/AKT pathway components [63]. These observations indicate that AMPs may either promote or inhibit EGFR signaling—along with other major pathways—depending on the physiological or pathological state of the target cells (healthy versus cancerous).

In some cases, AMPs induce cancer cell death through mechanisms bridging intrinsic and extrinsic apoptotic programs. The frog-skin peptide SSTP1 (also referred to as Temporin 1IDau1) exhibited selective cytotoxicity against human tongue squamous carcinoma HSC-4 cells, as shown by MTT assay, while sparing non-tumor cells [166]. Annexin V/PI staining revealed that SSTP1 markedly increased the proportion of cells in early apoptosis. This was supported by activation of caspase-3, -7, -9, and PARP cleavage, whereas caspase-8 remained unaffected, pointing to dominant involvement of mitochondrial (intrinsic) apoptosis.

RNA-sequencing analysis further demonstrated that SSTP1 significantly regulated TNF, JAK-STAT, and cytokine–receptor interaction pathways, suggesting participation of receptor-mediated signaling. Indeed, the peptide was shown to physically associate with the active IL-6/IL-6R/gp130 complex on the plasma membrane. This interaction selectively suppressed the pro-proliferative JAK/STAT cascade while simultaneously activating a pro-apoptotic IL-6/JNK/AP-1 pathway, revealing a complex network of signaling events converging on caspase-mediated apoptosis [166].

(iii) Autophagy-Related Pathway

The brevinin superfamily of peptides comprises the brevinin-1 and brevinin-2 groups, both known for broad-spectrum antimicrobial activity against diverse bacterial and fungal pathogens [167]. Within this family, brevinin-2R has demonstrated activity against *K. pneumoniae* [168] and *Leishmania* species [169].

Beyond its antimicrobial effects, brevinin-2R has emerged as a promising anticancer peptide. It exhibits semi-selective cytotoxicity toward multiple tumor cell lines, including T cell leukemia, B cell lymphoma, fibrosarcoma, breast and lung cancer cells, while exerting minimal or no toxicity toward peripheral blood mononuclear cells (PBMCs), normal T cells, or healthy human lung fibroblasts [59]. Remarkably, its cytotoxic potency exceeded that of standard chemotherapeutics such as doxorubicin and cisplatin in highly sensitive cancer models, including MCF-7 breast cancer and Jurkat T cell leukemia cells. Mechanistic analyses revealed that brevinin-2R does not induce caspase-dependent apoptosis. Co-treatment with a pan-caspase inhibitor failed to impair its cytotoxic effects, and no increase in expression or activation of caspase-3, -8, or -9 was detected following peptide exposure.

The mitochondrial pathway appears only partially involved in brevinin-2R-mediated cell death. In several tumor cell lines, treatment with this peptide triggered ROS generation and reduced ATP levels—hallmarks of mitochondrial dysfunction. Consistently, overexpression of the anti-apoptotic protein Bcl-2 markedly attenuated brevinin-2R cytotoxicity. However, because cell death proceeded independently of caspases, the authors examined the involvement of mitochondrial release of AIF and EndoG—key mediators of caspase-independent apoptosis [170]. Neither of these factors was released during brevinin-2R-induced cell death.

Given these observations, the authors investigated whether autophagy-associated lysosomal pathways contributed to cytotoxicity. Lethal signaling events can activate autophagy and promote cell death through lysosomal permeabilization and release of their lytic enzymes [171]. Indeed, brevinin-2R caused pronounced lysosomal swelling and permeabilization, accompanied by cytosolic translocation of cathepsin-B. The peptide also co-localized with both early and late endo-lysosomal markers, suggesting direct interaction with lysosomal membranes. Additional ultrastructural changes—including ER disintegration, cytoplasmic vacuolization, and autophagosome formation—further supported the involvement of autophagy.

Collectively, these findings indicate that brevinin-2R exerts its anticancer activity primarily through induction of autophagy and lysosome-mediated cell death, with a secondary contribution from partial activation of mitochondrial dysfunction [59].

(iv) Necroptosis Pathway

Necroptosis is a form of programmed cell death positioned at the interface between unregulated necrosis and caspase-dependent apoptosis. It is typically activated during chronic inflammatory conditions or viral infections. Central to this pathway are the RIPK3 kinase and its downstream effector MLKL, whose phosphorylation triggers membrane permeabilization and cell disruption [172].

Among amphibian AMPs, tigerinin-1 shows potent activity against *S. aureus* [173] and displays notable cytotoxicity toward various cancer cell types—including hepatoma, breast, lung, and prostate cancer cells. In particular, tigerinin-1 exhibits pronounced efficacy against A549 lung cancer cells [153]. In this cell line, the peptide reduces viability by promoting plasma membrane rupture and compromising lysosomal integrity, as demonstrated by LDH release and neutral red uptake (NRU) assays, respectively.

At the molecular level, tigerinin-1 selectively enhances the expression and phosphorylation of necroptosis-associated proteins RIP/p-RIP and MLKL/p-MLKL, while leaving levels of classical apoptotic markers (total or cleaved caspase-3 and -8) unchanged. These findings indicate that tigerinin-1 cytotoxic activity proceeds independently of apoptosis. Because necroptosis is frequently accompanied by elevated production of reactive oxygen species (ROS) [172], it is notable that tigerinin-1 induces a dose-dependent increase in ROS and related oxidative-stress markers—including lactoperoxidase (LPO), nitric oxide (NO), protein carbonyl (PC), and hydrogen peroxide (H_2_O_2_)—in lung cancer cells. This oxidative burden contributes to significant DNA damage, further compromising tumor cell viability.

Functionally, tigerinin-1 markedly reduces colony formation of A549 cells in soft agar, indicating inhibition of anchorage-independent growth. Moreover, when tested on HUVEC endothelial cells, the peptide suppresses capillaries formation, suggesting a potential angiostatic effect that may augment its antitumor activity [153].

### 2.2. Functional Consequences of AMP Treatment on Tumor Cells

For several of the anticancer AMPs listed in Table 1, the molecular mechanisms underlying their cytotoxic effects have not yet been fully elucidated. Nonetheless, a range of in vitro experimental studies has provided valuable insights into how these peptides functionally modulate tumor cell behavior. Several AMPs have been evaluated using different in vivo experimental tumor models, including syngeneic murine leukemia systems and human tumor xenografts in immunodeficient mice, highlighting the diversity of preclinical approaches used to assess their anticancer therapeutic potential.

#### 2.2.1. Regulation of Tumor Cell Proliferation, Migration, and Clonogenicity

The *Xenopus*-derived AMP magainin-2 and its two analogues—MSI-136 (“all-L”) and MSI-238 (“all-D”)—were initially tested in vitro on Ehrlich ascites tumor cells and A549 lung cancer cells using MTT assays. In both cell lines, the two analogues exhibited a more pronounced cytotoxicity compared with native magainin-2 [118].

To further evaluate their anticancer potential, the three peptides were tested in vivo in mouse models of leukemia, sarcoma, and spontaneous ovarian teratoma. In a syngeneic P388 leukemia ascites model in DBA/2 mice, where peptides were administered intraperitoneally, MSI-136 and MSI-238 modestly extended animal survival, though to a lesser extent than cisplatin. Magainin-2 produced a comparable effect only at higher doses. In syngeneic S180 sarcoma model, both analogues—but not magainin-2—achieved 100% increases in life span relative to untreated controls. The most compelling results came from studies in spontaneous ovarian teratoma in LT/Sv mice, where magainin-2 and its analogues significantly increased survival and delayed ascites tumor growth, reaching efficacy levels comparable to doxorubicin. Histopathological analyses showed that MSI-136 and MSI-238 also reduced tumor colonization of the peritoneum and mesenteric lymph nodes.

In terms of antimicrobial activity, magainin-2 is active against *S. aureus mprF* mutants [117] and *Batrachochytrium dendrobatidis* [20].

The AMPs dermaseptin-B2 and dermaseptin-B3, purified from *Phyllomedusa bicolor* skin extracts, were active against Gram-negative and Gram-positive bacteria [174] including *S. aureus*, *P. aeruginosa*, and *E. coli* [83]. Additionally, they have also shown significant anticancer activity, inhibiting PC-3 prostate cancer cell proliferation. They markedly suppressed anchorage-independent colony formation in soft agar, achieving effects comparable to 5-fluorouracil, and displayed anti-angiogenic activity in endothelial cell assays. Crucially, these antitumor effects were reproduced with synthetic versions of the peptides, emphasizing their drug-development potential [175].

The anticancer role of dermaseptin-B2 has been confirmed further in breast cancer cells [81]. In this study, MCF-7 tumor cells and non-tumor MCF-10A cells were transduced with a plasmid expressing dermaseptin-B2. Growth assays using crystal violet staining showed that dermaseptin-B2 expression significantly impaired MCF-7 proliferation while exerting only minor effects on MCF-10A cells. Consistently, tumor-specific reductions in colony formation in soft agar were observed.

Supporting these findings, wound-healing assays demonstrated a substantial inhibition of cell migration in dermaseptin-B2-expressing MCF-7 cells. Flow-cytometry analyses further revealed that dermaseptin-B2 expression selectively increased apoptotic susceptibility and induced G0/G1 cell-cycle arrest in tumor but not normal cells. At the molecular level, dermaseptin-B2 reduced the expression of anti-apoptotic genes, increased pro-apoptotic gene expression, and inhibited components of the pro-proliferative AKT1/AKT3 signaling pathway, highlighting its multi-level suppression of tumor growth and survival.

#### 2.2.2. Regulation of Immune Response

The AMP esculentin-2CHa and its double-substituted [D20K,D27K] analog exhibited antimicrobial activity against *S. aureus* and *E. coli*, and displayed dose-dependent cytotoxicity toward A549 lung cancer cells [176]. Beyond its antimicrobial and anticancer effects, esculentin-2CHA also shows immunomodulatory properties. When administered to mouse peritoneal macrophages, it did not alter IL-1β or IL-6 expression, either in the presence or absence of LPS stimulation. However, it significantly increased TNF-α expression compared with untreated controls and further enhanced TNF-α levels when combined with LPS. The peptide also modulated lymphoid cells, inducing a significant upregulation of the anti-inflammatory cytokine IL-10 alone or in combination with concanavalin A (known T cells mitogen), relative to their respective controls.

Two additional frog-skin-secretion-derived peptides, pseudohymenochirin-1Pb and pseudohymenochirin-2Pa, were examined for cytotoxicity in several tumor cell lines, including lung, metastatic breast, and colon cancer cells [131]. Among them, pseudohymenochirin-1Pb displayed selective, dose-dependent cytotoxicity toward cancer cells, whereas pseudohymenochirin-2Pa showed non-selective toxicity affecting non-tumor cells even at low doses. Their immunomodulatory potential was assessed in mouse macrophages: both peptides inhibited the expression of the anti-inflammatory cytokine IL-10 while increasing the production of the pro-inflammatory cytokine IL-23, including under LPS stimulation. Neither peptide affected IL-1β or TNF-α levels. Interestingly, IL-6—whose inflammatory role can vary with context [177]—was consistently downregulated. Both peptides displayed strong activity against Gram-positive bacteria, including multidrug-resistant clinical isolates, but showed only moderate effects against Gram-negative bacteria and *C. albicans* [131].

The peptides frenatin-2.1S and frenatin-2.2S also demonstrated potent antimicrobial activity against clinical isolates of MRSA and *S. epidermidis*, though they exhibited weaker activity against Gram-negative bacteria [103]. Both peptides displayed moderate cytotoxicity toward A549 lung cancer cells and were evaluated for immunomodulatory effects in mouse peritoneal macrophages. Consistent with trends observed for other frog-skin AMPs, frenatin-2.1S enhanced TNF-α expression in LPS-stimulated macrophages. Moreover, both frenatin-2.1S and frenatin-2.2S increased IL-1β and IL-23 levels in the presence of LPS. Notably, frenatin-2.2S downregulated IL-10 expression under LPS stimulation, while IL-6 remained unaffected by either peptide.

The antitumor and immunomodulatory potential of frenatin-2.1S was further investigated in vivo [104]. Although the peptide showed no effect on the viability of 4T1 mouse mammary carcinoma cells in vitro, as measured by real-time impedance-based cell index [178], its administration to a human A549 lung cancer xenograft model in BALB/c nude mice caused significant inhibition of tumor growth and markedly influenced immune-cell recruitment. Frenatin-2.1S increased the numbers of CD3^+^ T cells and CD11c^+^ dendritic cells in the peritoneal cavity. In addition, it elevated both the abundance and activation status of NK cells, as evidenced by upregulation of the cell surface markers NKG2D, FasL, CD69, and CD107a. Functionally, NK cells isolated from frenatin-2.1S-treated mice, but not peritoneal leukocytes, induced significant cytolysis of 4T1 cells in co-culture. These findings suggest that frenatin-2.1S exerts indirect antitumor effects by promoting the enrichment and activation of cytolytic NK cells [104].

### 2.3. Conservation of AMP Mechanisms of Action Across Species

Amphibian-derived AMPs represent a broad class of antimicrobial peptides whose anticancer mechanisms have been outlined in detail in the previous sections. The literature also contains abundant experimental data, compiled in both recent and earlier reviews, derived from studies of AMPs originating from a variety of other species, including humans, bovines, insects, and fish, as well as peptides of synthetic origin [28,179,180]. Notably, the mechanisms of action described for these dual-function peptides are largely comparable to those reported by us and others for amphibian AMPs [181].

These mechanisms frequently fall into several major categories, including membrane permeabilization or lysis; induction of apoptosis (via both intrinsic and extrinsic pathways); necrosis; cytolysis; regulation of the cell cycle; inhibition of angiogenesis; and modulation of immune responses. An exception is represented by certain amphibian AMPs, such as buforin, whose activity also involves mechanisms related to the inhibition of protein synthesis and interference with DNA/RNA replication [182].

Taken together, these observations suggest that the mechanisms underlying the dual antimicrobial and anticancer functions of many AMPs are largely conserved across species, highlighting their potential as a broad-spectrum defense strategy against both infections and tumor progression.

### 2.4. Translational Implications and Safety Concerns of Dual-Action Amphibian AMPs

AMPs with dual activity generally offer the advantage of being potentially applicable to cancers that are driven or promoted by bacterial infections. For instance, gastric cancer can arise as a consequence of infection with *H. pylori* [183], gallbladder cancer may be originated by infection with *S. typhi* [184] or bladder cancer can be caused by infection of different pathogens, including *Streptococcus*, *Anaerococcus*, *Aerococcus* and *Pseudomonas* [185]. Furthermore, the use of AMPs with dual functionality may be beneficial during anticancer therapies that render patients particularly susceptible to opportunistic infections, either due to prolonged hospitalization or as a result of therapy-induced immunosuppression [186,187].

As previously mentioned, the tumor microbiota plays an important role in tumor progression. Dual-function AMPs may therefore be employed to eliminate harmful bacteria that are part of the tumor-associated microbiota [188]. In addition, AMPs generally display a relatively low propensity to induce drug resistance and are reasonably biocompatible, including those of non-natural origin that are synthetically produced to confer increased specificity, improved tolerability, or other advantageous properties [189]. Importantly, they can also be used in combination with conventional anticancer drugs, potentially enhancing their therapeutic efficacy through synergistic effects [190,191].

The disadvantages represent the other side of the same coin. While dual-function AMPs may target harmful components of the microbiota and contribute to slowing tumor progression, they may also disrupt beneficial microbial communities, such as those of gut or cervical microbiota [192]. Moreover, they may pose risks to normal host cells, exhibiting cytotoxic and hemolytic effects or triggering excessive immune responses or inflammatory reactions [193,194].

An additional drawback is the relatively high peptide concentrations that are often required to achieve a significant therapeutic effect, also due to the high susceptibility to proteolytic degradation [195,196]. Intratumoral delivery of peptides, when feasible, is generally more efficient compared to systemic administration [197,198].

With regard to natural amphibian AMPs, at present there are relatively few clinical trials and limited studies employing relevant tumor models, although some investigations have been conducted using modified or enhanced amphibian peptides [199,200,201]. The modification of specific amino acid residues, the addition of functional groups, and/or the use of these peptides in combination with conventional anticancer drugs are likely to represent key strategies for maximizing the translational potential of dual-function antimicrobial peptides.

## 3. Concluding Remarks

Overall, analysis of the available literature indicates that amphibian-derived AMPs represent a promising and versatile class of anticancer candidates with clear translational potential. Although the body of work addressing this topic remains heterogeneous in terms of experimental approaches, tumor models, and analytical methodologies, the accumulated evidence consistently supports the notion that amphibian AMPs exert reproducible and biologically meaningful antitumor effects. Importantly, these effects converge on a limited number of conserved mechanisms, suggesting that AMP-based anticancer strategies could be rationally optimized for therapeutic development.

A more detailed analysis of the literature revealed an important structural feature of AMPs exhibiting both antimicrobial and antitumor activities: most of the peptides analyzed contain at least one α-helical segment within their sequence (see Table 1). It is well established that α-helical secondary structure is one of the factors that enhance the ability of AMPs to interact with and disrupt bacterial and/or mammalian cellular membranes [202]. This structural characteristic appears to be a common motif among anticancer amphibian AMPs and may therefore partly explain the mechanisms underlying their dual biological activity.

From a translational perspective, the preferential interaction of many amphibian AMPs with tumor cell membranes—largely attributed to their net positive charge and the altered lipid composition of cancer cell surfaces—offers a mechanistic basis for tumor selectivity. While membrane disruption and cytolysis have been reported for both cationic and anionic AMPs, membranolysis emerges as a recurrent and therapeutically relevant mechanism. Nonetheless, distinguishing between direct membrane damage and secondary membrane alterations arising from downstream cell death pathways remains challenging and highlights the need for standardized assays and time-resolved analyses in future preclinical studies.

Although the cationic nature of AMPs remains a valid parameter for predicting tumor selectivity, an important challenge for membranolytic peptides is to discriminate between genuine tumor selectivity and nonspecific cytotoxicity. This difficulty is largely attributable to the common practice of administering peptides either to bacterial strains or to in vitro cultures of cell lines, with cell viability typically used as the primary readout in both cases. Whenever possible, we sought to provide information regarding the effects of AMPs on normal versus cancer cells in order to assess whether tumor specificity was indeed supported by the available data.

Crucially, the prevailing anticancer activity of amphibian AMPs appears to rely on the activation of regulated cell death programs rather than nonspecific cytotoxicity. Comprehensive molecular investigations across multiple tumor cell lines have demonstrated that these peptides induce apoptosis via both intrinsic (mitochondrial) and extrinsic (death receptor-mediated) pathways, mechanisms that are already clinically exploited by several approved anticancer agents. The additional involvement of alternative cell death modalities, including autophagy and necroptosis, is of particular clinical relevance, as it suggests that AMPs may overcome resistance mechanisms associated with apoptosis-defective or therapy-refractory tumors.

Functionally, amphibian AMPs consistently suppress tumor cell proliferation, survival, and clonogenic expansion in vitro, including under three-dimensional and anchorage-independent growth conditions that more closely recapitulate in vivo tumor architecture. Their ability to impair tumor cell migration and motility further underscores their potential to limit invasion and metastatic spread—key determinants of poor clinical outcome. Although still limited in number, in vivo studies using mouse tumor models provide compelling proof-of-concept evidence that AMP treatment can reduce tumor growth and dissemination without overt systemic toxicity, thereby supporting continued preclinical development.

Beyond their direct effects on tumor cells, amphibian AMPs also exhibit immunomodulatory properties that may enhance their therapeutic value. Studies using immune cell cultures, particularly macrophages, indicate that anticancer AMPs promote a pro-inflammatory milieu by increasing the production of pro-inflammatory cytokines while suppressing anti-inflammatory signals. This dual activity—combining direct tumor cell killing with immune activation—positions amphibian AMPs as attractive candidates for multimodal cancer therapies, including combination strategies with chemotherapy, radiotherapy, or immune checkpoint inhibitors.

A crucial point is to determine to which extent the dual antimicrobial and anti-tumor activities of AMPs are truly coupled or simply converge on membrane-targeting mechanisms. Defining this would require specific experimental settings or in vivo models, because conventional 2D in vitro cultures of tumor cells fail to accurately reproduce the complexity of the in vivo tumor microenvironment, e.g., tissue organization, vascularization, immune infiltration, and the microbiota. This latter is a key regulator of tumor progression and therapeutic response [203].

Thus, a proof of concept of full functional duality would be identifying AMPs capable of concurrently suppressing tumor growth and bacterial proliferation in adequate oncology models where microbiota alterations and tumor progression are linked. This aspect, at least for amphibian AMPs, remains insufficiently explored.

Consequently, the lack of such specific studies makes it difficult to predict whether antimicrobial potency correlates with tumor selectivity. Notably, a substantial portion of the literature addressing the application of AMPs in oncology is based on synthetic derivatives that preserve the core structural features of AMPs—predominantly an α-helical conformation—while incorporating additional modifications designed to enhance tumor specificity [28]. Taken together, the available evidence supports amphibian-derived AMPs as promising leads for anticancer drug development. Future translational efforts should focus on improving peptide stability, bioavailability, and tumor selectivity, as well as on defining optimal delivery strategies and combination regimens. Addressing these challenges through rigorous preclinical and early-phase clinical studies will be essential to advance amphibian AMPs from experimental systems toward clinically viable anticancer therapeutics.

## Figures and Tables

**Table 1:** Frog-skin derived AMPs with dual anticancer (AC) and antibacterial (AB) activity.

Peptide Name and Sequence	Frog Species	Hydrophobic Residues (%) ^a^	3D Structure ^a^	AC Activity	AB Activity	Ref.
Alyteserin-2a ILGKLLSTAAGLLSNL.NH_2_	*Alytes obstetricans*	56	Helix	A549 (LC_50_ 80 µM) HepG2 (LC_50_ 85 µM) HT-29 (LC_50_ 80 µM) MDA-MB-231 (LC_50_ 65 µM)	*S. aureus* ATCC 25923 (MIC 64 µM) *E. coli* ATCC 25726 (MIC 256 µM) *K. pneumoniae* ATCC 700603 (MIC 256 µM) *P. aeruginosa* ATCC 27853 (MIC 256 µM) *A. baumannii* NM8 clinical isolate (MIC 64 µM) *S. maltophilia* B32/4 clinical isolate (MIC 128 µM) *S. epidermidis* RP62A biofilm-producer (MIC 64 µM) *S. epidermidis* RP62A/1 biofilm non-producer variant of RP62A (MIC 32 µM)	[31,32,33]
Ascaphin-8 GFKDLLKGAAKALVKTVLF.NH_2_	*Ascaphus truei*	57	Helix	HCT116 (IC_50_ 18.83 µM) MCF-7 (IC_50_ 9.33 µM) U87MG (IC_50_ 9.25 µM)	*E. coli* ATCC 25922 (MIC 6 µM) *P. aeruginosa* ATCC 27853 (MIC 13 µM) *E. cloacae* NHTCC 53001 (MIC 6 µM) *K. pneumoniae* KK3 9904 (MIC 6 µM) *S. epidermidis* RP62A biofilm producer (MIC 6 µM) *Streptococcus* Group B (MIC 6 µM) *E. faecalis* ATCC 29212 (MIC 50 µM) *S. aureus* NCTC 8325 (MIC 3–6 µM) MRSA USA 300 LAC (MIC 3.1 µM) *E. coli* K12 (MIC 12.5 µM) *B. subtilis* 168 (MIC 6.25 µM) *P. aeruginosa* PAO1 (MIC 12.5 µM)	[34,35,36]
Aurein 1.2 GLFDIIKKIAESF.NH_2_	*Ranoidea raniformis* (formerly *Litoria raniformis*)	53	Helix	Cancer type (LC_50_ in M): Breast (10^−5^) CNS (10^−5^) Colon (10^−5^) Leukaemia (10^−(4–5)^) Lung (10^−5^) Melanoma (10^−5^) Ovarian (10^−5^) Prostate (10^−5^) Renal (10^−5^)	*B. cereus* (MIC 100 μg/mL) * E. coli (MIC > 100 μg/mL) * L. lactis (MIC 12 μg/mL) * L. innocua (MIC 100 μg/mL) * M. luteus (MIC 100 μg/mL) * P. multocida (MIC 100 μg/mL) * S. aureus (MIC 50 μg/mL) * S. epidermidis (MIC 50 μg/mL) * S. uberis (MIC 50 μg/mL) *	[37,38,39]
Aurein 2.5 GLFDIVKKVVGAFGSL.NH_2_	*Ranoidea aurea* (formerly *Litoria aurea*); *Ranoidea raniformis* (formerly *Litoria raniformis*)	56	Helix	Cancer type (LC_50_ in M): Breast (10^−5^) CNS (10^−5^) Colon (10^−(4–5)^) Leukaemia (10^−(4–5)^) Lung (10^−5^) Melanoma (10^−(4–5)^) Ovarian (10^−5^) Prostate (10^−5^) Renal (10^−5^)	*B. cereus* (MIC 50 μg/mL) * E. coli (MIC > 100 μg/mL) * L. lactis (MIC 12 μg/mL) * L. innocua (MIC 50 μg/mL) * M. luteus (MIC 100 μg/mL) * P. multocida (MIC > 100 μg/mL) * S. aureus (MIC 50 μg/mL) * S. epidermidis (MIC 100 μg/mL) * S. uberis (MIC > 100 μg/mL) *	[38,40]
Aurein 2.6 GLFDIAKKVIGVIGSL.NH_2_	*Ranoidea raniformis* (formerly *Litoria raniformis*)	56	Helix	Cancer type (LC_50_ in M): Breast (10^−5^) CNS (10^−5^) Colon (10^−(4–5)^) Leukaemia (10^−(4–5)^) Lung (10^−5^) Melanoma (10^−5^) Ovarian (10^−5^) Prostate (10^−5^) Renal (10^−5^)	*B. cereus* (MIC 100 µg/mL) * E. coli (MIC > 100 µg/mL) * L. lactis (MIC 6 µg/mL) * L. innocua (MIC 100 µg/mL) * M. luteus (MIC 25 µg/mL) * P. multocida (MIC > 100 µg/mL) * S. aureus (MIC 50 µg/mL) * S. epidermidis (MIC 50 µg/mL) * S. uberis (MIC > 100 µg/mL) *	[38,39]
Aurein 3.1 GLFDIVKKIAGHIAGSI.NH_2_	*Ranoidea aurea* (formerly *Litoria aurea*); *Ranoidea raniformis* (formerly *Litoria raniformis*) *Ranoidea raniformis* (formerly *Litoria raniformis*)	52	Helix	Cancer type (LC_50_ in M): Breast (10^−(4–5)^) CNS (10^−(4–5)^) Colon (10^−5^) Leukaemia (10^−4^) Lung (10^−4^) Melanoma (10^−(4–5)^) Ovarian (10^−(4–5)^) Prostate (10^−5^) Renal (10^−(4–5)^)	*B. cereus* (MIC > 100 µg/mL) * E. coli (MIC > 100 µg/mL) * L. lactis (MIC 12 µg/mL) * L. innocua (MIC 100 µg/mL) * M. luteus (MIC 100 µg/mL) * P. multocida (MIC > 100 µg/mL) * S. aureus (MIC 50 µg/mL) * S. epidermidis (MIC 100 µg/mL) * S. uberis (MIC 100 µg/mL) *	[38,39]
Aurein 3.2 GLFDIVKKIAGHIASSI.NH_2_	*Ranoidea aurea* (formerly *Litoria aurea*); *Ranoidea raniformis* (formerly *Litoria raniformis*)	52	Helix	Cancer type (LC_50_ in M): Breast (10^−5^) CNS (10^−5^) Colon (10^−(4–5)^) Leukaemia (10^−5^) Lung (10^−5^) Melanoma (10^−5^) Ovarian (10^−5^) Prostate (10^−5^) Renal (10^−5^)	*B. cereus* (MIC > 100 µg/mL) * E. coli (MIC > 100 µg/mL) * L. lactis (MIC 6 µg/mL) * L. innocua (MIC 100 µg/mL) * M. luteus (MIC 100 µg/mL) * P. multocida (MIC > 100 µg/mL) * S. aureus (MIC 50 µg/mL) * S. epidermidis (MIC 50 µg/mL) * S. uberis (MIC 50 µg/mL) *	[38,39]
Aurein 3.3 GLFDIVKKIAGHIVSSI.NH_2_	*Ranoidea raniformis,* (formerly *Litoria raniformis*)	52	Helix	Cancer type (LC_50_ in M): Breast (>10^−5^) CNS (10^−5^) Colon (10^−5^) Leukaemia (10^−(4–5)^) Lung (10^−5^) Melanoma (10^−5^) Ovarian (10^−5^) Prostate (10^−5^) Renal (10^−5^)	*B. cereus* (MIC > 100 µg/mL) * E. coli (MIC > 100 µg/mL) * L. lactis (MIC 12 µg/mL) * L. innocua (MIC > 100 µg/mL) * M. luteus (MIC 100 µg/mL) * P. multocida (MIC 100 µg/mL) * S. aureus (MIC 100 µg/mL) * S. epidermidis (MIC 50 µg/mL) * S. uberis (MIC 25 µg/mL) *	[38,39]
Bombinin H4 LLGPVLGLVGSALGGLLKKI.NH_2_	*Bombina variegata*	55	Helix	A549 (n.d.) Calu-3 (n.d.)	*E. coli* D21 (LC 4.8 µM) *Y. pseudotuberculosis* YPIII (LC 8.2 µM) *E. agglomerans* Bo-1S (LC 11.3 µM) *B. megaterium* Bm11 (LC 0.8 µM) *S. aureus* Cowan I (LC 3.3 µM) *S. epidermidis* 1457 and 5179-R1 (n.d.) *S. lentus* (LC 2.0 µM) * M. luteus (LC 2.0 µM) * P. syringae pv *tabaci* (LC 8.2 µM) *	[41,42,43]
Bombinin-BO1 GIGSAILSAGKSIIKGLAKGLAEHF.NH_2_	*Bombina orientalis*	48	Helix	HepG2 (LC_50_ 3.75 µM) Huh7 (LC_50_ 3.91 µM) SK-HEP-1 (LC_50_ 0.76 µM)	*S. aureus* CPCC 100520 (MIC 26.3 µM) *E. coli* CPCC 100521 (MIC 26.3 µM)	[39,44]
Bombinin-like peptide 7 (BLP-7) GIGGALLSAGKSALKGLAKGLAEHFAN.NH_2_	*Bombina orientalis* also known as Maximin-6 from *Bombina variegata*	48	Helix	HepG2 (IC_50_ 2.83 µM) Huh7 (IC_50_ 3.87 µM) SK-HEP-1 (IC_50_ 0.61 µM) A375 (n.d.) A549 (n.d.) PC-3 (n.d.)	*S. aureus* CPCC 100520 (MIC 6.3 µM) *E. coli* CPCC 100521 (MIC 6.3 µM)	[39,45,46,47]
Brevinin-1-AW FLPLLAGLAANFLPQIICKIARKC	*Amolops wuyiensis*	67	C-terminal Cys-bridged cyclic domain, helix	H838 (LC_50_ 32.15 μM) PC-3 (LC_50_ 33.46 μM) U251MG (LC_50_ 33.56 μM)	*S. aureus* ATCC 6538 (MIC 32 µM) MRSA NCTC 12493 (MIC 32 µM) *E. faecalis* NCTC 12697 (MIC 32 µM) *E. coli* ATCC 8739 (MIC > 512 µM) *K. pneumoniae* ATCC 43816 (MIC > 512 µM) *P. aeruginosa* ATCC 9027 (MIC > 512 µM)	[39,48]
Brevinin-1BLa FLPAIVGAAAKFLPKIFCAISKKC	*Lithobates blairi*	66	C-terminal Cys-bridged cyclic domain, helix	HepG2 (LC_50_ 12 µM)	*E. coli* ATCC 25922 (MIC 25 µM) *S. aureus* ATCC 25923 (MIC 1.5 µM)	[39,49]
Brevinin-1BLc FLPIIAGIAAKFLPKIFCTISKKC	*Lithobates blairi*	62	C-terminal Cys-bridged cyclic domain, helix	HepG2 (LC_50_ 6 µM)	*E. coli* ATCC 25922 (MIC 25 µM) *S. aureus* ATCC 25923 (MIC 1.5 µM)	[39,49]
Brevinin-1BW FLPLLAGLAASFLPTIFCKISRKC	*Pelophylax nigromaculatus*	63	C-terminal Cys-bridged cyclic domain, helix	A375 (LC_50_ 23.79 µg/mL) A549 (LC_50_ 27.64 µg/mL) HCT116 (LC_50_ 35.44 µg/mL) HeLa (LC_50_ 37.62 µg/mL) HepG2 (LC_50_ 25.42 µg/mL) U20S (LC_50_ 39.20 µg/mL)	*S. aureus* ATCC 25923 (MIC 6.25 µg/mL) MRSA ATCC 29213 (MIC 6.25 µg/mL) *E. faecalis* ATCC 29212 (MIC 3.125 µg/mL) *S. saprophyticus* ATCC BAA750 (MIC 6.25 µg/mL) *S. epidermidis* CI 1607BL1D39 (MIC 12.5 µM) *S. haemolyticus* CI 1607BLD590 (MIC 12.5 µg/mL) *E. coli* ATCC 25922 (MIC 100 µg/mL) *E. hormaechei* ATCC700323 (MIC 100 µg/mL) *P. aeruginosa* ATCC 27853 (MIC 100 µg/mL) *K. pneumoniae* ATCC700603 (MIC > 100 µg/mL) *C. koseri* CI 1611SED223 (MIC 50 µg/mL)	[50]
Brevinin-1BYa FLPILASLAAKFGPKLFCLVTKKC	*Rana boylii*	62	C-terminal Cys-bridged cyclic domain, helix	A549 (IC_50_ 9.24 µM) C4-2B (IC_50_ 13.96 µM) Huh7 (IC_50_ 14.86 µM)	*E. coli* ATCC 25922 (MIC 17–20 µM) *K. pneumoniae* KK3 9904 (MIC 40 µM) *E. cloacae* HNTCC 53001 (MIC n.d.) *P. aeruginosa* ATCC 27853 (MIC 10 µM) *E. faecalis* ATCC 29212 (MIC 5 µM) *Streptococcus* Group B HNTCC 80130 (MIC 10 µM) *S. epidermidis* RP62A biofilm producer (MIC 5 µM) *S. aureus* NCTC 8352 (MIC 2 µM)	[51,52]
Brevinin-1DYb FLSLALAALPKLFCLIFKKC	*Rana dybowskii*; also known as amurin-2 from *Rana amurensis*	75	C-terminal Cys-bridged cyclic domain, helix	H157 (IC_50_ 4.5 µM) MCF-7 (IC_50_ 9.1 µM) MDA-MB-435S (IC_50_ 9.5 µM) PC-3 (IC_50_ 6.4 µM) U251MG (IC_50_ 4.6 µM)	*S. aureus* NCTC 10788 (MIC 32µM) MRSA ATCC 12493 (MIC 32 µM) *E. coli* NCTC 110418 (MIC > 512 µM) *E. coli* (MIC 15–32 µM) *	[53,54]
Brevinin-1EMb FLPLLAGLAANFLPTIICKISYKC	*Rana rugosa* also known as Gaegurin 6 from *Glandirana emeljanovi*	62	C-terminal Cys-bridged cyclic domain, helix	A549 (IC_50_ 6.49 μg/mL) Hek 293 (IC_50_ 5.62 μg/mL) Hep3B (IC_50_ 4.91 μg/mL) MCF-7 (IC_50_ 4.88 μg/mL) PC-3 (IC_50_ 5.75 μg/mL)	*M. luteus* ATCC 4698 (MIC 2.5 µg/mL) *S. epidermidis* ATCC 12228 (MIC 10 µg/mL) *B. subtilis* KCTC 1021 (MIC 10 µg/mL) *K. pneumoniae* ATCC 10031 (MIC 50 µg/mL) *S. dysenteriae* ATCC 9752 (MIC 50 µg/mL) *P. aeruginosa* ATCC 9027 (MIC 150 µg/mL) *E. coli* DH5 (MIC 25 µg/mL) *K. pneumoniae* ATCC 10031 (MIC 25 µg/mL) *S. typhimurium* ATCC 14028 (MIC 50 µg/mL) *E. coli* (MIC 100 µg/mL) *	[55,56]
Brevinin-1Ya FLPVIAGVAANFLPKLFCAISKKC	*Lithobates yavapaiensis*	66	C-terminal Cys-bridged cyclic domain, helix	HepG2 (LC_50_ 16 µM)	*E. coli* ATCC 25922 (MIC 50 µM) *S. aureus* ATCC 25923 (MIC 3 µM)	[49]
Brevinin-1Yb FLPIIAGAAAKVVQKIFCAISKKC	*Lithobates yavapaiensis*	66	C-terminal Cys-bridged cyclic domain, helix	HepG2 (LC_50_ 11 µM)	*E. coli* ATCC 25922 (MIC 25 µM) *S. aureus* ATCC 25923 (MIC 3 µM)	[49]
Brevinin-1Yc FLPIIAGAAAKVVEKIFCAISKKC	*Lithobates yavapaiensis*	66	C-terminal Cys-bridged cyclic domain, helix	HepG2 (LC_50_ 14 µM)	*E. coli* ATCC 25922 (MIC 100 µM) *S. aureus* ATCC 25923 (MIC 6 µM)	[49]
Brevinin-2KP GVITDALKGAAKTVAAELLKKAHCKLTNSC	*Kaloula pulchra*	50	Helix	(IC_50_ 3.27–59.75 μM): H460 H3122 Kyse-150 M21 MDA-MB-231 MDA-MB-435S Raw 264.7	*E. coli* ATCC 25922 (MIC 38.7 µM) *P. acnes* ATCC 6919 (MIC 25 µM) *S. aureus* ATCC 25923 (MIC > 100 µM) *B. subtilis* CMCC 63501 (MIC > 100 µM)	[57]
Brevinin-2R KLKNFAKGVAQSLLNKASCKLSGQC	*Pelophylax ridibundus*	44	C-terminal Cys-bridged cyclic domain, helix	BJAB (LD_50_ 30–40 μg/mL) Jurkat (LD_50_ 20–25 μg/mL) MCF-7 (LD_50_ 10–15 μg/mL) A549 (n.d.) HT29 (n.d.)	*S. aureus* 1112 (MIC 1.56 µg/mL) *E. coli* ATCC 2592 (MIC 0.39 µg/mL) *P. aeruginosa* ATCC 27853 (MIC 25 µg/mL) *K. pneumoniae* ATCC 13883 (MIC 12.5 µg/mL)	[58,59]
Caerin 1.1 GLLSVLGSVAKHVLPHVVPVIAEHL.NH_2_	*Pengyilleyia rothii* (formerly *Litoria rothii*); *Pelodryas splendida*, (formerly *Litoria splendida*)	56	Helix	(IC_50_~5–13 µg/mL): B16 MCF-7 SkBr-3 TC-1 A549 (IC_50_ 15.62 µg/mL) B-CPAP (IC_50_ 4.038 µg/mL) CAL-62 (IC_50_ 9.856 µg/mL) HeLa (IC_50_~10^−9^–10^−8^ M)	*S. aureus* ATCC 25923 or 29213 or MRSA (MIC 1–3 µg/mL) *B. cereus* (MIC 50 µg/mL) * E. coli (MIC > 100 µg/mL) * L. lactis (MIC 1.5 µM) * L. innocua (MIC 25 µg/mL) * M. luteus (MIC 12 µg/mL) * E. faecalis (MIC 25 µg/mL) * S. epidermidis (MIC 12 µg/mL) * S. uberis (MIC 25 µg/mL) * E. cloacae (MIC 12 µg/mL) * P. multocida (MIC 12–25 µg/mL) * P. haemolytica (MIC 25 µg/mL) *	[60,61,62,63,64,65,66]
Caerin 1.9 GLFGVLGSIAKHVLPHVVPVIAEKL.NH_2_	*Chlorohyla chloris* (formerly *Litoria chloris*)	54	Helix	(IC_50_ 5–13 µg/mL): B16 B-CPAP CAL-62 HeLa MCF-7 SkBr-3 TC-1 A549 (IC_50_ 18.52 µg/mL) TE-1 (IC_50_ 13.00 µg/mL) U87MG (IC_50_ 5.018 µg/mL) (Caerin 1.1 in combination with Caerin 1.9) U118MG (IC_50_ 11.180 µg/mL) (Caerin 1.1 in combination with Caerin 1.9)	*S. aureus* ATCC 25923 and 29213, or MRSA (MIC 3.75–12 µg/mL) *M. luteus* (MIC 50 µg/mL) * L. innocua (MIC 25–50 µg/mL) * S. epidermidis (MIC 25 µg/mL) * S. uberis (MIC 50 µg/mL) * B. cereus (MIC 100 µg/mL) * L. lactis (MIC 12 µg/mL) * S. hemolyticus (MIC 7.5 µg/mL) * A. baumannii (MIC 7.5 µg/mL) * E. coli (MIC 30 µg/mL) * P. aeruginosa (MIC 60 µg/mL) *	[61,62,63,64,67,68,69,70]
Citropin 1.1 GLFDVIKKVASVIGGL.NH_2_	*Dryopsophus citropa* (formerly *Litoria citropa*)	56	Helix	(IC_50_ 10^−5^ M): Breast CNS Colon Leukaemia Lung Melanoma Ovarian Prostate Renal U937 (IC_50_ 20 μg/mL)	*E. faecalis* PCM 2673 (MIC 32 µg/mL) *S. aureus* ATCC 25923 or 9144 (MIC 16 µg/mL) *S. pneumoniae* ATCC 49619 (MIC 32 µg/mL) *E. coli* ATCC 25922 or 23506 (MIC 32 µg/mL) *K. pneumoniae* ATCC 700603 (MIC 16 µg/mL) *P. aeruginosa* ATCC 9027 (MIC 128 µg/mL) *L. lactis* (MIC 3–6 µg/mL) * M. luteus (MIC 12–25 µg/mL) * S. epidermidis (MIC 12–25 µg/mL) * L. innocua (MIC 25–100 µg/mL) * S. uberis (MIC 12–25 µg/mL) * S. aureus or MRSA (MIC 25 µg/mL) * B. cereus (MIC 25–50 µg/mL) * B. subtilis (MIC 12.5 µg/mL) *	[71,72,73,74,75,76]
CPF-ST3 GLLGPLLKIAAKVGSNLL.NH_2_	*Silurana tropicalis* diploid clawed frog (formerly *Xenopus tropicalis)* (formerly described as peptide XT-7)	55	Helix	HepG2 (LC_50_ 75 µM)	*S. aureus* NCTC 8325 (MIC 5 µM) *S. aureus* 8325 clinical isolate (MIC 6 µM) MRSA 0470504 clinical isolate (MIC 7 µM) *S. epidermidis* 1420129 clinical isolate (MIC 3 µM) *S. saprophyticus* 1950556 clinical isoalate (MIC 13 µM) *E. coli* 25922 (MIC 6 µM) *Streptococcus* group C 1541035 (MIC 3 µM) *S. sonnei* 1840187 (MIC 35 µM) *P. aeruginosa* 0380972 clinical isolate (MIC 60 µM) *E. cloacae* 1441323 clinical isolate (MIC 35 µM)	[77,78,79]
Dermaseptin-DU-1 ALWKSLLKNVGKAAGKAALNAVTDMVNQ.NH_2_	*Callimedusa (Phyllomedusa) duellmani*	53	Helix	H157 (LC_50_ 8.43 µM) PC-3 (LC_50_ 21.6 µM)	*S. aureus* NCTC 10788 (MIC 4 µM) MRSA NCTC 12493 (MIC 4 µM) *E. faecalis* NCTC 12697 (MIC 64 µM) *K. pneumoniae* ATCC 43816 (MIC 4 µM) *P. aeruginosa* ATCC 27853 (MIC 4 µM) *E. coli* NCTC 10418 (MIC 4 µM)	[80]
Dermaseptin-B2 GLWSKIKEVGKEAAKAAAKAAGKAALGAVSEAV.NH_2_	*Phyllomedusa bicolor*	54	Helix	DU145 (LC_50_ 0.71–0.91 μM) HTK (LC_50_ 1.80 μM) LB-EBV (LC_50_ 1.09 μM) LNCaP (LC_50_ 0.31–2.65 μM) MDA-MB-231 (LC_50_ 8.06 μM) PC-3 (LC_50_ 1.24–2.17 μM) PNT1A (LC_50_ 0.38 μM) RAJI (LC_50_ 2.57 μM) HT-144 (LC_50_ 5.08 μM) A375 (LC_50_ > 10 μM) SK-MEL-28 (LC_50_ > 10 μM) PANC-1 (LC_50_ > 10 μM) MDA-MB-453 (LC_50_ > 10 μM) U87MG (LC_50_ > 10 μM) U138MG (LC_50_ > 10 μM) U373MG (LC_50_ > 10 μM) HT-29 (non-toxic)	*E. coli* IP 76-24 (MIC 15 μg/mL) *E. coli* 184 (MIC 7.5 μg/mL) *E. coli* ATCC8739 (MIC 3.75 μg/mL) *S. aureus* ATCC 25923 (MIC 0.7 μM) *P. aeruginosa* (MIC 3.1 μM) *	[81,82,83,84]
Dermaseptin-B3 ALWKNMLKGIGKLAGQAALGAVKTLVGA	*Phyllomedusa bicolor*	57	Helix	MCF-7 (n.d.) PC-3 (n.d.)	*S. aureus* ATCC 25923 (MIC 1.3 µM) *P. aeruginosa* CIP A22 (MIC 2.3 µM) *P. aeruginosa* ATCC 27853 (MIC 5.0 µM) *E. coli* ATCC 25922 (MIC 2.6 µM)	[39,83]
Dermaseptin-L1 GLWSKIKEAAKAAGKAALNAVTGLVNQGDQPS	*Agalychnis lemur* (formerly *Hylomantis lemur*)	43	Helix	HepG2 (LC_50_ 45 µM)	*E. coli* ATCC 25726 (MIC 8 µM)	[39,85]
Dermaseptin-PD-1 GMWSKIKETAMAAAKEAAKAAGKTISDMIKQ.NH_2_	*Pachymedusa dacnicolor*	48	Helix	U251MG (LC_50_ 15.08 μM)	*E. coli* NCTC 10418 (MIC 19.6 µM) *S. aureus* NCTC 10788 (MIC 39.2 µM) *P. aeruginosa* ATCC 27853 (MIC 19.6 µM)	[39,86]
Dermaseptin-PD-2 GMWSKIKNAGKAAAKAAAKAAGKAALDAVSEAI.NH_2_	*Pachymedusa dacnicolor*	57	Helix	H157 (LC_50_ 6.43 μM) PC-3 (LC_50_ 3.17 μM) U251MG (LC_50_ 13.43 μM)	*E. coli* NCTC 10418 (MIC 5 µM) *S. aureus* NCTC 10788 (MIC 5 µM) *P. aeruginosa* ATCC 27853 (MIC 2.5 µM)	[39,86]
Dermaseptin-PH ALWKEVLKNAGKAALNEINNLVQ	*Pithecopus* (*Phyllomedusa*) *hypochondrialis*	54	Helix	H157 (LC_50_ 2.01 µM) MCF-7 (LC_50_ 0.69 µM) MDA-MB-435S (LC_50_ 9.94 µM) PC-3 (LC_50_ 11.8 µM) U251MG (LC_50_ 2.36 µM)	*S. aureus* NCTC 10788 (MIC 32 µM) *E. faecalis* NCTC12697 (MIC 32 µM) MRSA NCTC 12493 (MIC > 512 µM) *E. coli* NCTC 10418 (MIC 16 µM) *P. aeruginosa* ATCC 27853 (MIC 64 µM)	[87]
Dermaseptin-PS1 ALWKTMLKKLGTVALHAGKAALGAVADTISQ.NH_2_	*Phyllomedusa sauvagii*	54	Helix	U251MG (LC_50_ 5.419 μM)	*S. aureus* NCTC 10788 (MIC 10 µM) *E. coli* NCTC 10418 (MIC 10 µM)	[39,88]
Dermaseptin-PS3 ALWKDILKNAGKAALNEINQIVQ	*Phyllomedusa sauvagii*	52	Helix	H157 (LC_50_ 15.67 µM) PC-3 (LC_50_ 18.2 µM)	*E. coli* NCTC 10418 (MIC 32 µM) *S. aureus* NCTC 10788 (MIC 256 µM)	[39,89]
Dermaseptin-PS4 ALWKTLLKHVGKAAGKAALNAVTDMVNQ.NH_2_	*Phyllomedusa sauvagii*	54	Helix	H157 (LC_50_ 0.19 µM) MCF-7 (LC_50_ 0.67 µM) MDA-MB-435S (LC_50_ 0.11 µM) PC-3 (LC_50_ 0.44 µM) U251MG (LC_50_ 57.66 nM)	*S. aureus* ATCC 12600 (MIC 4 µM) MRSA ATCC BAA1720 (MIC 8 µM) *E. faecalis* ATCC 29212 (MIC 32 µM) *E. coli* ATCC 11755 (MIC 8 µM) *P. aeruginosa* ATCC 27853 (MIC 16 µM)	[90]
Dermaseptin-PT9 GLWSKIKDAAKTAGKAALGFVNEMV	*Phyllomedusa tarsius*	52	Helix	H157 (LC_50_ 25.51 μM) MCF-7 (LC_50_ 7.44 μM) PANC-1 (LC_50_ 28.95 μM) PC-3 (LC_50_ 21.78 μM) U251MG (LC_50_ 49.51 μM)	*S. aureus* NCTC 10788 (MIC 16 µM) MRSA NCTC 12493 (MIC 32 µM) *E. faecalis* NCTC 12697 (MIC 16 µM) *E. coli* NCTC 10418 (MIC 8 µM) *P. aeruginosa* ATCC 27853 (MIC 16 µM) *K. pneumoniae* ATCC 43816 (MIC 8 µM)	[91]
Dermaseptin-SS1 ALWKSILKNAGKAALNEINQIV.NH_2_	*Phyllomedusa tarsius*	52	Helix	H460 (LC_50_ 1.3 µM) H838 (LC_50_ 7.7 µM)	*S. aureus* ATCC CRM 6538 (MIC 8 µM) MRSA NCTC 12493 (MIC > 128 µM) *E. faecalis* NCTC 12697 (MIC > 128 µM) *E. coli* ATCC CRM 8739 (MIC 2 µM) *E. coli* ATCC BAA2340 (MIC 4 µM) *E. coli* ATCC BAA2469 (MIC 4 µM) *E. coli* ATCC BAA2471 (MIC 8 µM) *E. coli* NCTC 13846 (MIC 8 µM) *K. pneumoniae* ATCC 43816 (MIC 16 µM) *P. aeruginosa* ATCC CRM 9027 (MIC 32 µM) *A. baumannii* ATCC BAA747 (MIC 16 µM)	[92]
Distinctin-Like-Peptide-PH NLVSALIEGRKYLKNVLKKLNRLKEKNKAKNSKENN	*Pithecopus* (*Phyllomedusa*) *hypochondrialis*	30	Helix	H157 (LC_50_ 15.41 µg/mL) PC-3 (LC_50_ 32.25 µg/mL)	*S. aureus* NCTC 10788 (MIC 256 µg/mL) *E. coli* NCTC 10418 (MIC 32 µg/mL) *P. aeruginosa* ATCC 27853 (MIC 64 µg/mL)	[93]
Dybowskin-2 FLIGMTQGLICLITRKC	*Rana dybowskii*; *Rana chensinensis*	58	Helix	HeLa (n.d.) MCF-7 (n.d.)	*M. luteus* ATCC 4698 (MIC 3.3 µM) *S. aureus* KCTC 3881 (MIC 7.9 µM) *B. subtilis* ATCC 6633 (MIC 13.1 µM) *S. epidermidis* ATCC 12228 (MIC 13.1 µM) *S. dysenteriae* ATCC 9752 (MIC 26.2 µM) *E. coli* KCTC 2433 (MIC 31.4 µM) *K. pneumoniae* ATCC 10031 (MIC 31.4 µM) *P. mirabilis* ATCC 25933 (MIC > 52.4 µM) *P. aeruginosa* ATCC 9027 (MIC > 52.4 µM)	[94,95]
Dybowskin-3 GLFDVVKGVLKGVGKNVAGSLLEQLKCKLSGGC	*Rana dybowskii* also known as Brevinin-2RNa isolated from *Pelophylax nigromaculatus* (formerly *Rana nigromaculata*)	45	C-terminal Cys-bridged cyclic domain, helix	HeLa (IC_50_ 50.2 µM) MCF-7 (IC_50_ 49.3 µM)	*M. luteus* ATCC 4698 (MIC 1.9 µM) *S. aureus* KCTC 3881 (MIC 9.0 µM) *B. subtilis* ATCC 6633 (MIC 15.1 µM) *S. epidermidis* ATCC 12228 (MIC 18.1 µM) *S. dysenteriae* ATCC 9752 (MIC 18.1 µM) *E. coli* KCTC 2433 (MIC 4.5 µM) *K. pneumoniae* ATCC 10031 (MIC 4.5 µM) *P. mirabilis* ATCC 25933 (MIC > 30.2 µM) *P. aeruginosa* ATCC 9027 (MIC > 30.2 µM) *E. coli* AS1.349 (MIC > 100 µM) *S. aureus* AS1.72 (MIC 37.5 µM) *B. cereus* (MIC 37.5 µM) * P. aeruginosa (MIC > 100 µM) * S. lactis (MIC 37.5 µM) *	[94,95,96]
Esculentin-2 CHa GFSSIFRGVAKFASKGLGKDLAKLGVDLVACKISKQC	*Lithobates chiricahuensis*	48	C-terminal Cys-bridged cyclic domain, helix	A549 (LC_50_ 10 µM)	*S. aureus* ATCC 25923 (MIC 5 µM) MRSA 127/08, 145/08, 274/08, V4180, S908, T4/6 clinical isolates (MIC 6 µM) *E. coli* ATCC 25726 (MIC 6 µM) *P. aeruginosa* ATCC 27853 (MIC 5 µM) *K. pneumoniae* ATCC 700603 (MIC 5 µM) *A. baumannii* NM8, NM75, NM35, NM109, NM124 clinical isolates (MIC 6 µM) *S. maltophilia* B32/1, B32/4, B5/5, B6/2, U8708 clinical isolates (MIC 3–6 µM)	[97]
Esculentin-2 HYba1 SIFSLFKMGAKALGKTLLKQAGKAGAEYAACKATNQC	*Hydrophylax bahuvistara*	49	Helix	Hep3B (n.d.)	*S. aureus* MTCC 9542 (MIC 3 µM) MRSA ATCC 43300 (MIC 5 µM) *B. subtilis* MTCC 14416 (MIC 10 µM) *B. coagulans* ATCC 7050 (MIC 15 µM) *E. faecalis* ATCC 29212 (MIC 12 µM) *S. mutans* MTCC 497 (MIC 15 µM) *S. gordonnii* MTCC 2695 (MIC 15 µM) *V. cholerae* MCV 09 (MIC 9 µM) *E. coli* ATCC 25922 (MIC 12 µM)	[98]
Esculentin-2 HYba2 SILSLFKMGAKALGKTLIKQAGKAGAEYVACKATNQC	*Hydrophylax bahuvistara*	49	C-terminal Cys-bridged cyclic domain, helix	Hep3B (n.d.)	*S. aureus* MTCC 9542 (MIC 3 µM) MRSA ATCC 43300 (MIC 6 µM) *B. subtilis* MTCC 14416 (MIC 15 µM) *B. coagulans* ATCC 7050 (MIC 10 µM) *E. faecalis* ATCC 29212 (MIC 18 µM) *S. mutans* MTCC 497 (MIC 15 µM) *S. gordonnii* MTCC 2695 (MIC 10 µM) *V. cholerae* MCV 09 (MIC 10 µM) *E. coli* ATCC 25922 (MIC 15 µM)	[98]
Figainin 1 FIGTLIPLALGALTKLFK.NH_2_	*Boana raniceps*	61	Helix	B16-F10 (IC_50_ 10.5 µM) HeLa (IC_50_ 11.1 µM) MCF-7 (IC_50_ 13.7 µM)	*E. faecalis* ATCC 29212 (MIC 8 µM) *S. aureus* ATCC 25923 (MIC 4 µM) *S. epidermidis* ATCC 12228 (MIC 2 µM) *E. casseliflavus* ATCC 700327 (MIC 16 µM) *E. coli* ATCC 25922 (MIC 16 µM) *K. pneumoniae* ATCC 13883 (MIC 4 µM)	[99]
Figainin 2 FLGAILKIGHALAKTVLPMVTNAFKPKQ	*Boana raniceps*	53	Helix	B16-F10 (LC_50_ 12.8 µM) MCF-7 (LC_50_ 15.3 µM)	*S. aureus* ATCC 25923 (MIC 8 µM) *E. faecalis* ATCC 29212 (MIC 8 µM) *S. epidermidis* ATCC 12228 (MIC 4 µM) *E. casseliflavus* ATCC 700327 (MIC 4 µM) *E. coli* ATCC 25922 (MIC 8 µM) *K. pneumoniae* ATCC 13883 (MIC 8 µM) *K. pneumoniae* KPC MR (MIC 16 µM) *P. aeruginosa* ATCC 27853 (MIC 32 µM)	[100]
Figainin 2BN FLGVALKLGKVLGKALLPLASSLLHSQ	*Boana boans*	56	Helix	A549 (LC_50_ 6.7 µM) HT-29 (LC_50_ 14.1 µM) MDA-MB-231 (LC_50_ 8.1 µM)	*S. aureus* ATCC 12600 (MIC 7.8 µM) *E. faecium* ATCC 19439 (MIC 31.3 µM) *E. faecalis* ATCC 51299 (MIC 125 µM) *E. coli* ATCC 35218 (MIC 15.6 µM) *K. pneumoniae* ATCC 49472 (MIC 31.3 µM) *P. aeruginosa* ATCC 9072 (MIC 31.3 µM)	[101]
Figainin 2PL FLGTVLKLGKAIAKTVVPMLTNAMQPKQ	*Boana platanera*	50	Helix	A549 (LC_50_ 6.4 µg/mL) HT-29 (LC_50_ 19.2 µg/mL) MDA-MB-231 (LC_50_ 7.0 µg/mL)	*E. coli* DSM 787 (MIC 6.25 µg/mL) *S. aureus* ATCC 43300 (MIC 6.25 µg/mL) *S. epidermidis* DSM 28319 (MIC 12.5 µg/mL) *P. aeruginosa* DSM 50071 (MIC 100 µg/mL) *K. pneumoniae* ATCC BAA1705 (MIC 25 µg/mL) *A. baumannii* DSM 30008 (MIC 6.25 µg/mL) *E. faecalis* MF 06036 (MIC 50 µg/mL) *E. faecium* NCTC 12201 (MIC 6.25 µg/mL) *C. difficile* R20291 or 630 BAA1382 (MIC 6.25 µg/mL)	[102]
Frenatin 2.1S GLVGTLLGHIGKAILG	*Sphaenorhynchus lacteus*	50	Helix	A549 (LC_50_ 80–140 µM) 4T1 (n.d.)	*S. aureus* ATCC 25293 (MIC 8 µM) MRSA 127/08, 145/08, 274/08, S908, T4/6, V4180 clinical isolates (MIC 8–16 µM) *S. epidermidis* RP62A biofilm producer, RP62A/1 biofilm non-producer variant of RP62A, T6/19 and T37/8 clinical isolates (MIC 8–16 µM) *E. coli* ATCC 25726 (MIC 125 µM) *K. pneumoniae* ATCC 700603 (MIC 250 µM) *P. aeruginosa* ATCC 27853 (MIC 250 µM) *E. faecalis* ATCC 29212 (MIC 62.5 µM) *A. baumannii* NM8, NM35, NM75, NM109, NM124 clinical isolates (MIC 62.5–125 µM) *S. maltophilia* B32/1, B32/4, B5/5, B6/2, U8708 clinical isolates (MIC 31.3–62.5 µM) *X. axonopodis* pv. *vesicatoria* (MIC 3.1–6.2 µM) *	[103,104]
Frenatin 2.2S GLVGTLLGHIGKAILS	*Sphaenorhynchus lacteus*	50	Helix	A549 (LC_50_ 65 µM)	*S. aureus* ATCC 25293 (MIC 16 µM) MRSA 127/08, 145/08, 274/08, S908, T4/6, V4180 clinical isolates (MIC 8–16 µM) *S. epidermidis* RP62A biofilm producer, RP62A/1 biofilm non-producer variant of RP62A, T6/19 and T37/8 clinical isolates (MIC 8–16 µM) *A. baumannii* NM8, NM35, NM75, NM109, NM124 clinical isolates (MIC 62.5–125 µM) *S. maltophilia* B32/1, B32/4, B5/5, B6/2, U8708 clinical isolates (MIC 31.3–62.5 µM) *E. coli* ATCC 25726 (MIC 125 µM) *K. pneumoniae* ATCC 700603 (MIC 250 µM) *P. aeruginosa* ATCC 27853 (MIC 250 µM) *E. faecalis* ATCC 29212 (MIC 62.5 µM) *X. axonopodis* pv. *vesicatoria* (MIC 6.2–12.5 µM) * X. arboricola pv. *pruni* (MIC 6.2–12.5 µM) *	[103]
Hylin PL FLGLIPALAGAIGNLIK	*Boana platanera*	65	Helix	A549 (LC_50_ 58 µg/mL) HT-29 (LC_50_ 68 µg/mL) MDA-MB-231 (LC_50_ 58 µg/mL)	*E. coli* DSM 787 (MIC 100 µg/mL) *S. aureus* ATCC 43300 (MIC 100 µg/mL) *S. epidermidis* DSM 28319 (MIC > 100 µg/mL) *P. aeruginosa* DSM 50071 (MIC > 100 µg/mL) *K. pneumoniae* ATCC BAA1705 (MIC > 100 µg/mL) *A. baumannii* DSM 30008 (MIC 100 µg/mL) *E. faecalis* MF 06036 (MIC 100 µg/mL) *E. faecium* NCTC 12201 (MIC 50 µg/mL) *C. difficile* R20291 or 630 BAA1382 (MIC 25–50 µg/mL)	[102]
Hymenochirin-1B IKLSPETKDNLKKVLKGAIKGAIAVAKMV	*Hymenochirus boettgeri*	48	Helix	A549 (LC_50_ 2.5 µM) HepG2 (LC_50_ 22.5 µM) HT-29 (LC_50_ 9.7 µM) MDA-MB-231 (LC_50_ 9.0 µM) H460 (n.d.) H1299 (n.d.) PLC (n.d.)	*E. coli* ATCC 25726 (MIC 20–25 µM) *P. aeruginosa* ATCC 27853 (MIC 40–50 µM) *K. pneumoniae* ATCC 700603 (MIC 20–25 µM) *S. aureus* ATCC 25923, ATCC 29213 (MIC 10–12.5 µM) *S. epidermidis* RP62A biofilm-producer (MIC 3.1 µM) *S. epidermidis* RP62A/1 biofilm non-producer variant of RP62A (MIC 1.6 µM)	[105,106]
Hymenochirin-1Pa LKLSPKTKDTLKKVLKGAIKGAIAIASMA	*Pseudhymenochirus merlini*	48	Helix	A549 (LC_50_ 1.2 µM) HT-29 (LC_50_ 12.3 µM) MDA-MB-231 (LC_50_ 3.1 µM)	*E. coli* ATCC 25726 (MIC 5–10 µM) *K. pneumoniae* ATCC 700603 (MIC 10 µM) *S. epidermidis* RP62A biofilm producer, RP62A/1 biofilm non-producer variant of RP62A, T7/3, T6/19 and T37/8 clinical isolates (MIC 0.6–1.25 µM) *S. aureus* 25923 (MIC 2.5 µM) MRSA 127/08, 145/08, 274/08, S908, T4/6, V4180 clinical isolates (MIC 1.25–2.5 µM) *E. faecalis* ATCC 29212 (MIC 20 µM) *P. aeruginosa* ATCC 27853 (MIC 20 µM) *A. baumannii* NM8, NM35, NM75, NM109, NM124 clinical isolates (MIC 2.5–5 µM) *S. maltophilia* B32/1, B32/4, B5/5, B6/2, U8708 clinical isolates (MIC 1.25–5 µM)	[107,108]
Kassiniatuerin-3 FIQHLIPLIPHAIQGIKDIF.NH_2_	*Kassina senegalensis*	55	Helix	H23 (LC_50_ 4.52 μM) H157 (LC_50_ 21.54 μM) H460 (LC_50_ 1.67 μM) H838 (LC_50_ 10.03 μM) LNCaP (LC_50_ 3.79 μM) U251MG (LC_50_ 15.20 μM)	*S. aureus* NCTC 10788 (MIC 16–32 µM) MRSA NCTC 12493 (MIC 32–64 µM) *E. faecalis* NCTC 12697 (MIC 128 µM) *E. coli* NCTC 10418 (MIC > 512 µM) *P. aeruginosa* ATCC 27853 (MIC > 512 µM)	[109]
*Limnonectes fujianensis* Brevinin (LFB) GLFSVVKGVLKGVGKNVSGSLLDQLKCKISGGC	*Limnonectes fujianensis*	42	C-terminal Cys but no disulfide bridge	H460 (LC_50_ 3.47 μM) HCT116 (LC_50_ 2.02 μM) MDA-MB-435S (LC_50_ 18.99 μM) U251MG (LC_50_ 2.32 μM)	*S. aureus* NCTC 10788 (MIC 16 µg/mL) *E. coli* NCTC 10418 (MIC 32 µg/mL)	[110]
Maculatin 1.1 GLFVGVLAKVAAHVVPAIAEHF.NH_2_	*Spicicalyx genimaculata* (formerly *Litoria genimaculata*); *Spicicalyx eucnemis* (formerly *Litoria eucnemis*)	67	Helix	(LD_50_ 10^–5^–10^–6^ M): Breast CNS Colon Leukaemia Lung Melanoma Ovarian Prostate Renal MCF-7 (IC_50_ 23 µM)	*L. lactis* (MIC 25 µg/mL) * S. uberis (MIC 3 µg/mL) * S. aureus (MIC 6–12 µg/mL) * M. luteus (MIC 12.5 µg/mL) * S. epidermidis (MIC 12.5 µg/mL) * S. faecalis (MIC 25 µg/mL) * B. cereus (MIC 25–50 µg/mL) * P. multocida (MIC 25–100 µg/mL) * E. coli (MIC > 100 µg/mL) * L. innocua (MIC 100 µg/mL) *	[70,111,112,113,114,115]
Maculatin 1.3 GLLGLLGSVVSHVVPAIVGHF.NH_2_	*Spicicalyx eucnemis* (formerly *Litoria eucnemis*)	57	Helix	Breast (n.d.) CNS (n.d.) Colon (n.d.) Leukaemia (n.d.) Lung (n.d.) Melanoma (n.d.) Ovarian (n.d.) Prostate (n.d.) Renal (n.d.)	MRSA USA300 (MIC 6.2 µM) *B. subtilis* 168 (MIC 25 µM) *E. coli* K12 (MIC > 100 µM) *P. aeruginosa* PAO1 (MIC > 100 µM)	[36,70]
Maculatin 1.4 GLLGLLGSVVSHVLPAITQHL.NH_2_	*Spicicalyx eucnemis* (formerly *Litoria eucnemis*)	52	Helix	Breast (n.d.) CNS (n.d.) Colon (n.d.) Leukaemia (n.d.) Lung (n.d.) Melanoma (n.d.) Ovarian (n.d.) Prostate (n.d.) Renal (n.d.)	*B. cereus* (MIC 100 μg/mL) * L. lactis (MIC 6 μg/mL) * L. innocua (MIC 100 μg/mL) * M. luteus (MIC 50 μg/mL) * S. aureus (MIC 50 μg/mL) * S. epidermidis (MIC 50 μg/mL) * S. uberis (MIC 50 µg/mL) * E. coli (MIC > 100 µg/mL) * P. multocida (MIC > 100 µg/mL) *	[70]
Magainin 2 GIGKFLHSAKKFGKAFVGEIMNS	*Xenopus laevis*	43	Helix	A549 (IC_50_ 110 µg/mL) Ehrlich ascites (n.d.) P388 (n.d.) S180 ascites (n.d.)	*E. coli* D31 (MIC 5 µg/mL) *K. pneumoniae* (MIC 10 µg/mL) * P. putida (MIC 10 µg/mL) * S. epidermidis (MIC 10 µg/mL) * C. freundii (MIC 30 µg/mL) * E. cloacae (MIC 50 µg/mL) * E. coli (MIC 50 µg/mL) * S. aureus (MIC 50 µg/mL) * P. aeruginosa (MIC 100 µg/mL) * S. marcescens (MIC 100 µg/mL) * P. mirabilis (MIC > 100 µg/mL) * S. fecalis ((MIC > 100 µg/mL) *	[19,116,117,118]
Maximin 1 GIGTKILGGVKTALKGALKELASTYAN.NH_2_	*Bombina maxima*	40	Helix	BIU-87 (IC_50_ 20.5 µg/mL) C8166 (IC_50_ 15.3 µg/mL) Molt-4 (IC_50_ 24.3 µg/mL) T24 (IC_50_ 35.4 µg/mL)	*E. coli* ATCC 25922 (MIC 19.5 µg/mL) *S. aureus* ATCC 25923 (MIC 19.5 µg/mL) *B. pyocyaneus* CMCCB 10104 (MIC 19.5 µg/mL) *B. megaterium* (MIC 19.5 µg/mL) * B. dysenterium (MIC 2.7 µM) * K. pneumoniae (MIC 9 µg/mL) *	[119]
Maximin 3 GIGGKILSGLKTALKGAAKELASTYLH	*Bombina maxima*	40	Helix	BIU-87 (IC_50_ 28 µg/mL) C8166 (IC_50_ 11.4 µg/mL) Molt-4 (IC_50_ 25.2 µg/mL) T24 (IC_50_ 18 µg/mL)	*E. coli* ATCC 25922 (MIC 0.9 µg/mL) *S. aureus* ATCC 2592 (MIC 3.1 µg/mL) *B. pyocyaneus* CMCCB 10104 (MIC 1.5 µg/mL) *B. megaterium* (MIC 0.9 µg/mL) * B. dysenterium (MIC 0.9 µg/mL) * K. pneumoniae (MIC 3.1 µg/mL) *	[119]
Maximin 4 GIGGVLLSAGKAALKGLAKVLAEKYAN.NH_2_	*Bombina maxima*	51	Helix	C8166 (IC_50_ 24.2 µg/mL) Molt-4 (IC_50_ 35.4 µg/mL) BIU-87 (IC_50_ > 50 µg/mL) T24 (IC_50_ > 50 µg/mL)	*E. coli* ATCC 25922 (MIC 2.7 µM) *S. aureus* ATCC 2592 (MIC 2.7 µM), *B. pyocyaneus* CMCCB 10104 (MIC 2.7 µM) *B. megaterium* (MIC 1.5 µM) * B. dysenterium (MIC 2.7 µM) * K. pneumoniae (MIC 15 µM) *	[119]
Maximin 5 SIGAKILGGVKTFFKGALKELASTYLQ	*Bombina maxima*	44	Helix	C8166 (IC_50_ 34.4 µg/mL) BIU-87 (IC_50_ > 50 µg/mL) Molt-4 (IC_50_ > 50 µg/mL) T24 (IC_50_ > 50 µg/mL)	*S. aureus* ATCC 2592 (MIC 3.6 µM) *B. pyocyaneus* CMCCB 10104 (MIC 7.2 µM) *B. megaterium* (MIC 12 µM) * B. dysenterium (MIC 12 µM) * K. pneumoniae (MIC 3.6 µM) *	[119]
Maximin H5 ILGPVLGLVSDTLDDVLGIL.NH_2_	*Bombina maxima*	55	Helix	T98G (IC_50_ 125 µM)	*S. aureus* ATCC 2592 (MIC 80 µM) *S. aureus* UL12 (MIC 90 µM)	[120,121,122]
Nigrocin-PN GLLGKILGAGKKVLCGVSGLC	*Pelophylax nigromaculatus*	52	C-terminal Cys-bridged cyclic domain, helix	H157 (LC_50_ 18.4 μM) PC-3 (LC_50_ 53.03 μM) U251MG (LC_50_ 73.65 μM)	*E. coli* NCTC 10418 (MIC 8 µM) *K. pneumoniae* ATCC 13883 (MIC 16 µM) *K. pneumoniae* ATCC 43816 (MIC 16 µM) *P. aeruginosa* ATCC 27853 (MIC 64 µM) *P. aeruginosa* ATCC 9097 (MIC 64 µM) *P. aeruginosa* B004 V2S2B (MIC 16 µM)	[123]
Ocellatin-3N GIFDVLKNLAKGVITSLAS.NH_2_	*Leptodactylus nesiotus*	52	Helix	A549 (LC_50_ 35 µM) MDA-MB-231 (LC_50_ 51 µM) HT-29 (LC_50_ 69 µM)	*S. aureus* ATCC 12600 (MIC 31.25 µM) *S. aureus* ATCC 25923 (MIC 12.5 µM) MRSA ATCC BAA2312 (MIC 31.25 µM) *E. faecium* ATCC 19434 (MIC 62.5 µM) *E. faecalis* ATCC 51299 (MIC 250 µM) *S. epidermidis* ATCC 12228 (MIC 6.25 µM) *B. megaterium* BM11 (MIC 0.78 µM) *E. coli* ATCC 35218 (MIC 31.25 µM) *E. coli* ATCC 25922 (MIC 6.25 µM) *K. pneumoniae* ATCC 49472 (MIC 62.5 µM) *K. pneumoniae* ATCC BAA2814 (MIC 62.5 µM) *P. aeruginosa* ATCC 27853 (MIC 25 µM) *A. baumannii* ATCC 19606 (MIC 6.25 µM) *S. typhimurium* ATCC 14028 (MIC 62.5 µM)	[124,125]
Palustrin-Ca GFLDIIKDTGKEFAVKILNNLKCKLAGGCPP	*Lithobates catesbeianus*	45	Helix	BEL7402 (LC_50_ 1.375 µg/mL) HeLa (LC_50_ 1.202 µg/mL) MGC803 (LC_50_ 1.572 µg/mL) SGC7901 (LC_50_ 0.951 µg/mL) SMMC7721 (LC_50_ 1.077 µg/mL)	*K. pneumoniae* ATCC 700603 (MIC 60 µg/mL) *P. aeruginosa* ATCC 227853 (MIC 30 µg/mL) *L. monocytogenes* ATCC 54004 (MIC 30 µg/mL) *S. aureus* ATCC 25923 (MIC 7.8 µg/mL) *S. suis* 2 CVCC 606 (MIC 31.25 µg/mL) *B. subtilis* ADB 403 (MIC 30 µg/mL)	[126]
Pentadactylin GLLDTLKGAAKNVVGSLASKVMEKL.NH_2_	*Leptodactylus pentadactylus*, *Leptodactylus labyrinthicus*	48	Helix	B16-F10 (IC_50_ 25.7 µM)	*E. coli* ATCC 25922 (MIC 25 µM) *E. cloacae* HNTCC 53001 (MIC 50 µM) *E. faecalis* ATCC 29212 (MIC 200 µM) *K. pneumoniae* KK3 9904 (MIC 100 µM) *P. aeruginosa* ATCC 27853 (MIC 100 µM) *P. mirabilis* ATCC 25933 (MIC > 200 µM) *S. aureus* NCTC 8325 (MIC 200 µM) *Streptococcus* Group B HNTCC 80130 (MIC 50 µM) *S. epidermidis* RP62A biofilm producer (MIC 100 µM)	[127,128]
Phylloseptin-L1 LLGMIPLAISAISALSKL.NH_2_	*Agalychnis lemur* (formerly *Hylomantis lemur*)	66	Helix	HepG2 (LC_50_ 35 µM)	*S. aureus* ATCC 25923 (MIC 8 µM)	[85]
Phylloseptin-PHa FLSLIPAAISAVSALANHF.NH_2_	*Pithecopus hypochondrialis* (formerly *Phyllomedusa hypochondrialis*)	68	Helix	H157 (LC_50_ 14.10 μM) HCT116 (LD_50_ 36.64 μM) MCF-7 (LD_50_ 24.05 μM) MDA-MB-435S (LD_50_ 28.48 μM) PC-3 (LD_50_ 37.96 μM) U251MG (LD_50_ 34.67 μM)	*S. aureus* NCTC 10788 (MIC 32.97 µM) MRSA NCTC 12493 (MIC 32.97 µM) *E. faecalis* NCTC 12697 (MIC 263.78 µM) *E. coli* NCTC 10418 (MIC > 263.78 µM)	[129,130]
Phylloseptin-PTa FLSLIPKIAGGIAALAKHL.NH_2_	*Phyllomedusa tarsius*	63	Helix	H157 (LC_50_ 6.73 μM)	*S. aureus* NCTC 10788 (MIC 4.14 µM) MRSA NCTC 10788 (MIC 4.14 µM) *E. faecalis* NCTC 12697 (MIC 16.56 µM) *E. coli* NCTC 10418 (MIC 16.56 µM)	[130]
Picturin 1BN GIFKDTLKKVVAAVLTTVADNIHPK	*Boana boans*	48	Helix	A549 (IC_50_ 30.7 µM) MDA-MB-231 (IC_50_ 64.2 µM) HT-29 (IC_50_ 84.9 µM)	*S. aureus* ATCC 12600 (MIC 62.5 µM) *E. coli* ATCC 35218 (MIC 7.8 µM) *K. pneumoniae* ATCC 49472 (MIC 15.6 µM) *P. aeruginosa* ATCC 9072 (MIC 15.6 µM)	[101]
Picturin 2BN GLMDMLKKVGKVALTVAKSALLP	*Boana boans*	57	Helix	A549 (IC_50_ 23 µM) MDA-MB-231 (IC_50_ 26.3 µM) HT-29 (IC_50_ 52.2 µM)	*S. aureus* ATCC 12600 (MIC 31.3 µM) *E. coli* ATCC 35218 (MIC 7.8 µM) *K. pneumoniae* ATCC 49472 (MIC 15.6 µM) *P. aeruginosa* ATCC 9072 (MIC 15.6 µM)	[101]
Pseudhymenochirin-1Pb IKIPSFFRNILKKVGKEAVSLIAGALKQS	*Pseudhymenochirus merlini*	48	Helix	A549 (LC_50_ 2.5 μM) MDA-MB-231 (LC_50_ 6.6 μM) HT-29 (LC_50_ 9.5 μM)	*S. aureus* ATCC 25923 (MIC 5 µM) *E. coli* ATCC 25726 (MIC 10 µM) *K. pneumoniae* ATCC 700603 (MIC 20 µM) *P. aeruginosa* ATCC 27853 (MIC 20 µM) *E. faecalis* ATCC 29212 (MIC 10 µM) MRSA 127/08, 145/08, 274/08, V4180, S908, T4/6 clinical isolates (MIC 5–10 µM) *A. baumannii* strains NM8, NM35, NM75, NM109, NM124 clinical isolates (MIC 5–10 µM) *S. maltophilia* B32/1, B32/4, B5/5, B6/2, and U8708 clinical isolates (MIC 5–10 µM) *S. epidermidis* RP62A biofilm producer, RP62A/1 biofilm non-producer variant of RP62A, T6/19 and T37/8 clinical isolates (MIC 1.25–2.5 µM)	[107,131]
Pseudhymenochirin-2Pa GIFPIFAKLLGKVIKVASSLISKGRTE	*Pseudhymenochirus merlini*	48	Helix	A549 (LC_50_ 6 μM) MDA-MB-231 (LC_50_ 6.2 μM) HT-29 (LC_50_ 11.5 μM)	*S. aureus* ATCC 25923 (MIC 5 µM) *E. coli* ATCC 25726 (MIC > 80 µM) *K. pneumoniae* ATCC 700603 (MIC > 80 µM) *P. aeruginosa* ATCC 27853 (MIC > 80 µM) *E. faecalis* ATCC 29212 (MIC 20 µM) MRSA 127/08, 145/08, 274/08, V4180, S908, T4/6 clinical isolates (MIC 5–10 µM) *A. baumannii* strains NM8, NM35, NM75, NM109, NM124 clinical isolates (MIC 10–20 µM) *S. maltophilia* B32/1, B32/4, B5/5, B6/2, and U8708 clinical isolates (MIC 10–40 µM) *S. epidermidis* RP62A biofilm producer, RP62A/1 biofilm non-producer variant of RP62A, T6/19 and T37/8 clinical isolates (MIC 2.5–10 µM)	[107,131]
Ranalexin FLGGLIKIVPAMICAVTKKC	*Lithobates catesbeianus* (formerly *Rana catesbeiana*)	65	C-terminal Cys-bridged cyclic domain, helix	HeLa (IC_50_ 13.4 µg/mL) COS7 (IC_50_ 14.8 µg/mL)	MRSA NCTC ATCC 10442 (MIC 8 µg/mL) *E. faecalis* ATCC 31299 (MIC 32 µg/mL) *S. pyogenes* ATCC 12344 (MIC 8 µg/mL) *K. pneumoniae* ATCC 700603 (MIC 128 µg/mL) *E. coli* ATCC 25922 (MIC 32 µg/mL) MRSA BC ATCC 210301 (MIC 16 µg/mL) MRSA KL ATCC 215947 (MIC 8 µg/mL) *A. baumanii* ATCC BAA747 (MIC 64 µg/mL) *A. baumanii* SR201346 (MIC 32 µg/mL) *S. aureus* or MRSA (MIC 4 µg/mL) *P. aeruginosa* (MIC 128 µg/mL) * B. megaterium DSM 32 (MIC 1.9 µM) *B. subtilis* DSM 10 (MIC 1.9 µM) *C. pasterianum* DSM 525 (MIC 7.6 µM) *C. spheniscorum* DSM 44757 (MIC 7.6 µM) *E. casseliflavus* ATCC 700327 VanC (MIC 3.8 µM) *E. faecalis* ATCC 29212 (MIC 7.6 µM) *E. faecium* UL407074 VanA 3 clinical isolate (MIC 7.6 µM) *S. aureus* ATCC 25923 (MIC 3.8 µM) MRSA NCTC 10442 (MIC 3.8 µM) *S. epidermidis* ATCC 14990 (MIC 7.6 µM) *S. saprophyticus* ATCC 15305 (MIC 3.8 µM) *A. baumannii* SC3033362 4-MRGN, SC3223332 4-MRGN, SC411190 4-MRGN clinical isolates (MIC 1.9–3.8 µM) *E. coli* ATCC 25922 (MIC 15.2 µM) *E. coli* 0157:H7 ATCC 35150 EHEC (MIC 15.2 µM) *K. pneumoniae* ATCC 700603 (MIC > 30.4 µM) *K. pneumoniae* BL809453 (MIC > 30.4 µM) *P. aeruginosa* ATCC 27853 (MIC 30.4 µM) *P. fluorescens* DSM 50090 (MIC > 30.4 µM) *Y. mollaretii* DSM 18520 (MIC > 30.4 µM)	[132,133]
Ranatuerin-2PLx GIMDTVKNAAKNLAGQLLDKLKCSITAC	*Rana palustris*	50	C-terminal Cys-bridged cyclic domain, helix	H157 (LC_50_ 5.9 μM) MCF-7 (LC_50_ 20.19 μM) MDA-MB-435S (LC_50_ 15.44 μM) PC-3 (LC_50_ 5.79 μM) U251MG (LC_50_ 16.14 μM)	*S. aureus* NCTC 10788 (MIC 32 µM) *E. coli* NCTC 10418 (MIC 32 µM) *E. faecalis* NCTC 12697 (MIC 128 µM) MRSA ATCC 12493 (MIC 256 µM) *P. aeruginosa* ATCC 27853 (MIC > 512 µM)	[134]
Ranatuerin-2Ya GLMDTIKGVAKTVAASWLDKLKCKITGC	*Lithobates yavapaiensis*	50	C-terminal Cys-bridged cyclic domain, helix	HepG2 (LC_50_ 20 μM)	*E. coli* ATCC 25922 (MIC 50 µM) *S. aureus* ATCC 25923 (MIC 50 µM)	[49]
Raniseptin PL GVFDTVKKIGKAVGKFALGVAKNYLNS	*Boana platanera*	44	Helix	A549 (IC_50_ 5.8 μg/mL) HT-29 (IC_50_ 29.1 μg/mL) MDA-MB-231 (IC_50_ 10.5 μg/mL)	*E. coli* DSM 787 (MIC 3.13 µg/mL) *S. aureus* ATCC 43300 (MIC 25 µg/mL) *S. epidermidis* DSM 28319 (MIC 6.25 µg/mL) *P. aeruginosa* DSM 50071 (MIC 50 µg/mL) *K. pneumoniae* ATCC BAA1705 (MIC 3.13 µg/mL) *A. baumannii* DSM 30008 (MIC 3.13 µg/mL) *E. faecalis* MF 06036 (MIC 25 µg/mL) *E. faecium* NCTC 12201 (MIC 3.13 µg/mL) *C. difficile* R20291 or 630 BAA1382 (MIC 3.13 µg/mL)	[102]
Raniseptin-3 AWLDKLKSIGKVVGKVAIGVAKNLLNPQ	*Boana raniceps*	50	Helix	B16-F10 (IC_50_ 6.56 μM) NIH3T3 (IC_50_ 4.21 μM)	*E. coli* ATCC 25922 (MIC 2 µM) *K. pneumoniae* ATCC 13883 (MIC 1 µM) *K. pneumoniae* ATCC 13883 (MIC 4 µM) *S. aureus* ATCC 25923 (MIC 4 µM) *S. epidermidis* ATCC 12228 (MIC 8 µM)	[135]
Raniseptin-6 ALLDKLKSLGKVVGKVALGVVQNYLNPRQ	*Boana raniceps*	45	Helix	B16-F10 (IC_50_ 8.69 μM)	*E. coli* ATCC 25922 (MIC 2 µM) *K. pneumoniae* ATCC 13883 (MIC 1 µM) *K. pneumoniae* ATCC 13883 (MIC 4 µM) *S. aureus* ATCC 25923 (MIC 32 µM) *S. epidermidis* ATCC 12228 (MIC 8 µM)	[135]
t-DPH1 GLWSKIKNVAAAAGKAALGAL	*Phyllomedusa hypochondrialis*	62	Helix	H838 (IC_50_ 23.51 μM) H157 (IC_50_ 10.20 μM) PC-3 (IC_50_ 14.67 μM) U251MG (IC_50_ 34.25 μM)	*S. aureus* ATCC 6538 or MRSA NCTC 12493 (MIC 8–16 µM) *E. faecalis* NCTC 12697 (MIC 128 µM) *E. coli* ATCC 8739 (MIC 2 µM) *K. pneumoniae* ATCC 43816 (MIC 8 µM) *P. aeruginosa* ATCC 9027 (MIC 16 µM)	[136]
Temporin A FLPLIGRVLSGIL.NH_2_	*Rana temporaria*	61	Helix	(IC_50_ 5.637 × 10^−7^ M): A549 Calu-3	*B. megaterium* Bm11 (LC 1.2 µM) *S. aureus* Cowan 1 (LC 2.3 µM) *E. coli* D21 (LC 11.9 µM) *A. hydrophila* Bo-3N (LC > 360 µM) *P. aeruginosa* ATCC15692 (LC > 360 µM) *C. jeikeium* ATCC BAA 949 (MIC 8 µM) *S. aureus* (MIC~3–8 µM) * E. coli (MIC 6.2–100 µM) * A. baumannii (MIC 24 µM) * Y. pseudotuberculosis (LC 2.0 µM) * S. pyogenes beta heme Group A (MIC 1–2.3 µM) *	[43,137,138]
Temporin L FVQWFSKFLGRIL.NH_2_	*Rana temporaria*	61	Helix	(IC_50_ 5.637 × 10^−7^ M): A549 Calu-3	*B. megaterium* Bm11 (LC 0.3 µM) *S. aureus* Cowan I (LC 0.5 µM) *E. coli* D21 (LC 1.5–12 µM) *E. coli* D21 e7 (LC 1.2 µM) *E. coli* D21 f1 (LC 0.9 µM) *E. coli* D21 f2 (LC 0.5 µM) *E. coli* D22 (LC 0.7 µM) *P. aeruginosa* ATCC 15692 (LC 3.6 µM) *Y. pseudotuberculosis* YP III (LC 0.7 µM) *S. capitis* 1 * (LC 0.7 µM) *S. capitis* 3 * (LC 0.3 µM) *S. hemolyticus* 1 * (LC 0.4 µM) *S. lentus* 1 * (LC 0.2 µM) *S. pyogenes* ATCC 12344 (LC 0.6 µM) *S. aureus* 7 * (LC 0.5 µM) *S. aureus* 8 * (LC 0.6 µM) *S. epidermidis* 11 * (LC 0.6 µM) *S. epidermidis* 18 * (LC 0.3 µM) *S. hominis* (LC 0.4 µM) * M. luteus (LC 0.3 µM) * P. aeruginosa (LC 17.0 µM) * S. aureus ATCC 25923 (MIC 3 µM)	[138,139]
Temporin-1CEa FVDLKKIANIINSIF.NH_2_	*Rana chensinensis*	60	Helix	A549 (LC_50_ 53.31 µM) Bcap-37 (LC_50_ 39.42 µM) BEL7402 (LC_50_ 38.98 µM) BGC-823 (LC_50_ 63 µM) H446 (LC_50_ 67.66 µM) HeLa (LC_50_ 36.31 µM) HO-8910 (LC_50_ 64.75 µM) HT-29 (LC_50_ > 100 µM) LK-2 (LC_50_ 63.4 µM) MCF-7 (LC_50_ 31.91 µM) MDA-MB-231 (LC_50_ 57.94 µM) SMMC7721 (LC_50_ 50.32 µM)	*E. amylovora* (MIC 25–50 µM) * *X. arboricola* pv. *pruni* (MIC 3.1–6.2 µM) * *X. axonopodis* pv. *vesicatoria* (MIC 3.1–6.2 µM) * S. aureus 22401 (MIC 14.4 µM) *E. coli* 44102 (MIC > 100 µM) *B. cereus* (MIC 14.4 µM) * S. lactis (MIC 14.4 µM) *	[140,141,142,143]
Temporin-1CEb ILPILSLIGGLLGK	*Rana chensinensis* also known as amurin-3 from *Rana amurensis*	57	Helix	A549 (n.d.) Bcap-37 (n.d.) BEL7402 (n.d.) BGC-823 (n.d.) H446 (n.d.) HeLa (n.d.) HO-8910 (n.d.) HT-29 (n.d.) LK-2 (n.d.) MCF-7 (n.d.) MDA-MB-231 (n.d.) SMMC7721 (n.d.)	*S. aureus* ATCC 22401 (MIC 41 µM) *E. coli* ATCC 44102 (MIC > 100 µM) *B. cereus* (MIC 41 µM) * S. lactis (MIC 41 µM) *	[144,145]
Temporin-1Oc FLPLLASLFSRLF.NH_2_	*Rana ornativentris*	69	Helix	4T1 (LC_50_ 65 µM) HeLa (LC_50_ 50 µM)	*S. aureus* NCTC 8325 (MIC 2 µM) *S. aureus* USA 300 (MIC 1.6 µM) *S. epidermidis* 1457 (MIC 1.6 µM) *B. subtilis* 168 (MIC 12.5 µM) *E. coli* ATCC 25922 (MIC > 150 µM) *K. pneumoniae* ATCC 13883 (MIC > 50 µM) * P. aeruginosa PAO1 (MIC > 50 µM)	[146,147]
Temporin-1RNb FLPLKKLRFGLL.NH_2_	*Rana nigromaculata*	58	Helix	HeLa (LC_50_ 12.8 µM) MCF-7 (LC_50_ 11.4 µM)	*S. aureus* AS1.72 (MIC 3.13 µM) *E. coli* AS1.349 (MIC 12.5 µM) *P. aeruginosa* (MIC 12.5 µM) * B. cereus (MIC 3.13 µM) * S. lactis (MIC 3.13 µM) *	[96]
Temporin-La LLRHVVKILEKYL.NH_2_	*Lithobates catesbeianus*	53	Helix	BEL7402 (LC_50_ 2.670 µg/mL) HeLa (LC_50_ 4.685 µg/mL) MGC803 (LC_50_ 3.937 µg/mL) SGC7901 (LC_50_ 2.755 µg/mL) SMMC7721 (LC_50_ 1.384 µg/mL)	*E. coli* ATCC 25922 (MIC > 100 µg/mL) *Salmonella* ATCC 20020 (MIC 15.6 µg/mL) *P. aeruginosa* ATCC 227853 (MIC 60 µg/mL) *K. pneumoniae* ATCC 700603 (MIC > 100 µg/mL) *S. aureus* ATCC 25923 (MIC 2.5 µg/mL) *S. suis* type 2 CVCC 606 (MIC 15.6 µg/mL)	[126,148]
Temporin-PE FLPIVAKLLSGLL.NH_2_	*Pelophylax kl. esculentus* (formerly *Rana esculenta*)	69	Helix	H157 (LC_50_ 34.56 µM) MDA-MB-435S (LC_50_ 33.23 µM) PC-3 (LC_50_ 38.56 µM) U251MG (LC_50_ 25.13 µM)	*S. aureus* NCTC 10788 (MIC 2 µM) MRSA NCTC 12493 (MIC 4 µM) *E. faecalis* NCTC 12697 (MIC 8 µM) *E. coli* NCTC 10418 (MIC 16 µM) *P. aeruginosa* ATCC 27853 (MIC 128 µM)	[149]
Temporin-SHf FFFLSRIF.NH_2_	*Pelophylax saharicus*	75	Helix	Cytotoxicity at 24 h A549 (IC_50_~24 µM) HepG2 (IC_50_~33 µM) MCF-7 (IC_50_~33 µM) PC-3 (IC_50_~37 µM)	*E. coli* ATCC 25922 (MIC 25 µM) *P. aeruginosa* ATCC 15442 (MIC 108 µM) *K. pneumoniae* ATCC 13883 (MIC 43 µM) *S. aureus* MTCC 9542 (MIC 34 µM) *E. faecalis* ATCC 29212 (MIC 50 µM) *B. subtilis* ATCC 6051 (MIC 39 µM) *P. aeruginosa* ATCC 27853 (MIC > 200 µM) *S. aureus* ATCC 25923 (MIC 12.5 µM) *E. faecalis* ATCC 29212 (MIC 50 µM) MRSA (MIC 25 µM) * B. megaterium (MIC 3 µM) *	[150,151]
Tigerinin 1 FCTMIPIPRCY.NH_2_	*Hoplobatrachus tigerinus* (formerly *Rana tigerina*)	54	Cys-containing, β-turn structure stabilized by a disulfide bridge	A549 (IC_50_~30 µM) HepG2 (IC_50_~40 µM) MCF-7 (IC_50_~30 µM) PC-3 (IC_50_~50 µM)	*M. luteus* MT 166 (MIC 20 µg/mL) *E. coli* W160-37 (MIC 10 µg/mL) *S. aureus* ATCC 8530 (MIC 10 µg/mL) MRSA clinical strain (MIC 50 µg/mL) *E. coli* clinical strains (MIC 25–35 µg/mL) *V. cholerae* clinical strain (MIC 120 µg/mL) *P. aeruginosa* clinical strain (MIC 150 µg/mL) *K. pneumoniae* clinical strain (MIC 50 µg/mL) *S. flexneri* clinical strain (MIC 70 µg/mL) *P. mirabilis* clinical strain (MIC 150 µg/mL) *S. enterica typhi* H clinical strain (MIC 45 µg/mL)	[152,153]
XLAsp-P1 *Xenopus laevis* antibacterial peptide-P1 DEDDD	*Xenopus laevis*	0	Unknown	MCF-7 (LC_50_ < 5 μg/mL)	*S. aureus* ATCC 29213 (MIC 10 µg/mL) *E. coli* ATCC 25922 (MIC 50 µg/mL) *S. sciuri* (MIC 50 µg/mL) * R. nasimurium (MIC 10 µg/mL) * A. viridans (MIC 20 µg/mL) * K. pneumoniae (MIC 50 µg/mL) * A. bacillus (MIC 50 µM) *	[154]

^a^ Data are taken either from the Antimicrobial Peptide DatabaseADP6 [39] or from the respective publications; *Human tumor-derived cancer cells*: **A549, H23, H157, H460, H838, H1299, H3122**, non-small cell lung cancer; **H446**, small cell lung cancer; **Calu-3**, lung epithelial cancer cells; **LK-2**, lung squamous cell carcinoma; **BEL7402, Hep3B, HepG2, Huh7, PLC, SK-HEP-1, SMMC7721**, hepatic cancer; **T98G, U87MG, U118MG, U138MG, U251MG**, **U373MG**, glioblastoma; **A375, HT-144, M21, MDA-MB-435S, SK-MEL-28**, melanoma; **U20S**, osteosarcoma; **HTK**, osteomyeoloma; **MCF-7, MDA-MB-231, MDA-MB-453, SkBr-3**, breast cancer; **Bcap-37**, breast cancer (problematic cell line, shown to be rather HeLa derivative); **HeLa**, cervical cancer; **HO-8910**, ovarian cancer; **C4-2B, DU145, LNCaP, PC-3, PNT1A**, prostate cancer; **Kyse-150, TE-1**, esophageal cancer; **B-CPAP, CAL-62**, thyroid cancer; **BGC-823, MGC803, SGC7901**, gastric cancer; **PANC-1**, pancreatic cancer; **HT-29, HCT116**, colon cancer; **Hek 293**, transformed kidney cell line; **BIU-87, T24**, bladder cancer; **Jurkat**, T-cell leukemia; **C8166, Molt-4**, T-cell cancer; **RAJI**, **BJAB**, B-cell lymphoma; **U937**, histiocytic lymphoma; **LB-EBV**, B-cells infected and immortalized by the Epstein–Barr virus (EBV); **HSC-4**, oral squamous cell carcinoma. *Non-human cancer cells*: **Raw 264.7**, **P388**, murine leukemic monocyte macrophage cells; **TC-1**, mouse cervical cancer; **B16, B16-F10**, murine melanoma; **4T1**, mouse mammary carcinoma; **NIH3T3**, mouse fibroblasts cells; **Ehrlich** ascites murine carcinoma; **S180**, ascites murine sarcoma; **COS7**, transformed African green monkey epithelial kidney cells. *—specific bacterial strain not indicated, **MRSA**—Methicillin-resistant *Staphylococcus aureus*, **MIC**—minimum inhibitory concentration, **LC**—lethal concentration, **IC_50_ or LC_50_**—concentration inhibiting 50% of cancer cell growth, **n.d.**—specific IC_50_ or MIC was not determined but the peptide was active against the cancer cells or the bacteria.

## Data Availability

No new data were created or analyzed in this study. Data sharing is not applicable to this article.
